# Supplementary material for: Covalently bridging graphene edges for improving mechanical and electrical properties of fibers
Source: Nat Commun. 2024 Jun 7;15:4880. doi: 10.1038/s41467-024-49270-5 (PMC11161649; doi:10.1038/s41467-024-49270-5)
Supplement: Supplementary file 1 — Supplementary Information [file 41467_2024_49270_MOESM1_ESM.pdf]

Supplementary Information

Covalently Bridging Graphene Edges for Improving Mechanical and Electrical Properties of Fibers

Ling Ding,[1†] Tianqi Xu,[1,2†] Jiawen Zhang,[1,2†] Jinpeng Ji,[1†] Zhaotao Song,[1,2†] Yanan Zhang,[1] Yijun Xu,[3] Tong Liu,[3] Yang Liu,[3] Zihan Zhang,[4] Wenbin Gong,[5] Yunong Wang,[1] Zhenzhong Shi,[1] Renzhi Ma,[4] Jianxin Geng,[2,6] Huynh Thien Ngo,[4] Fengxia Geng,[1,7*] Zhongfan Liu[7,8]

[1] College of Energy; School of Physical Science and Technology & Institute for Advanced Study, Soochow University, Suzhou 215006, China

[2] Beijing University of Chemical Technology, Beijing 100029, China

[3] Suzhou Institute of Nano-Tech and Nano-Bionics, Chinese Academy of Sciences, Suzhou, 215123, China

[4] National Institute for Materials Science, Tsukuba, Ibaraki, 305-0044, Japan

[5] School of Physics and Energy, Xuzhou University of Technology, Xuzhou, China

[6] State Key Laboratory of Separation Membranes and Membrane Processes; School of Material Science and Engineering, Tiangong University, Tianjin 300387, China

[7] Beijing Graphene Institute, Beijing 100095, China

[8] Peking University, Beijing 100871, China

† These authors contributed equally

E-mail: gengfx@suda.edu.cn

Supplementary discussion 1: Mechanical property, density, and electrical conductivity measurements

Tensile strength testing was conducted using a commercial mechanical tensile testing system (HY-0350, Shanghai Hengyi Precision Instrument Co., Ltd) equipped with a fine force detector with 0.00001 N accuracy following a previously described procedure.[1] Individual fibers were fixed with glue onto a hollow rectangular paper frame, and mounted between two clamp stages onto the testing system. The paper was cut prior to the testing experiments. Uniaxial tension on the samples was then applied with the top clamp stage along the vertical direction. Special care may be needed to ensure that the fibers fixed with glue remain tightly attached and the tension is uniaxially applied on the fiber during the tensile test. The gauge length in the tensile tests was 10 mm, and various loading strain rates including 0.01, 0.05, and 0.1 mm min^{-1} were tested. Adjusting these parameters in the above-mentioned range did not significantly affect the mechanical property measurement. The mechanical strength was calculated by dividing the fracture force by the fractural cross-sectional area, which was estimated from SEM images using ImageJ software. Young's modulus was determined from the mechanical stress and strain at breakage (%). Elongation was monitored using the tester software. To check the reproducibility of the results, the mechanical properties of the fibers were replicated for at least 10 samples for each set of experimental conditions.

The density of each fiber sample was tested using the sink-float method, in which two types of liquids, tetrabromoethane ($Br_2CHCHBr_2$, density 2.967 g cm^{-3}) and carbon tetrachloride (CCl_4, density 1.595 g cm^{-3}), were used as density markers. The fibers used for density testing were cut into small 5 mm long pieces. The sample density was taken to be equal to that of the liquid mixture in which the sample could be stably suspended for >4 h.

Electrical conductivity (σ) was measured using a standard four-probe method. Four equally spaced collinear silver probes were connected to the same side of the fiber sample using silver paste. A Keithley 2400 multiple-function source meter was used as the current source to apply a current across the two end probes, and the corresponding voltage change across the two inner probes was measured. σ (S m^{-1}) was calculated based on the relation $\sigma = \frac{LI}{SU}$, where I (A) is the applied current, U (V) is the

corresponding voltage, S (m^2) is the cross-sectional area measured by SEM, and L (m) is the distance between the probes (10 mm).

Supplementary discussion 2: Characterization

The liquid crystalline nature or mesoscopic order of the GO sheets and the oriented structure of the spun fibers were characterized using polarized optical microscopy (Olympus, IX73). Atomic force microscopy (AFM) (OXFORD, MFP-3D Origin) images were obtained using an ASYLUM RESEARCH instrument in tapping mode. The morphology and microstructure of the fibers were characterized by field-emission scanning electron microscopy (SEM) (Hitachi, SU8010). Scanning Transmission Electron Microscopy in an SEM (STEM-in-SEM, FEI Scios, acceleration voltage 30 kV) was employed to check the alignment of sheets over large areas. The microstructures of the graphene fibers were examined at the atomic scale using transmission electron microscopy (TEM) (FEI, Talos F200, acceleration voltage 200 kV). The electron-transparent sample slices used for TEM were cut using a Ga ion beam (6.4 nA beam current) on a focused ion beam microscope (FEI Scios) and transferred to a Cu ring holder.

Powder X-ray diffraction (XRD) patterns were recorded on a Bruker D8 Advance diffractometer using Cu-Kα radiation at a scanning speed of 3° min^{-1}. Wide-angle X-ray scattering (WAXS) of the filament bundles was carried out on a Xenocs Xeuss instrument to record two-dimensional diffractograms. The fibers were measured as aligned multifilament bundles in the transmission test mode. The degree of orientation relative to the fiber axis was quantified using the Herman's orientation factor, which is defined by the expression: $f = (\frac{3}{2}\cos^2\varphi - \frac{1}{2})$, where $\cos^2\varphi$ is the average value of the square of the cosine of the azimuthal angle (φ) of the (002) peak, given by the equation $\cos^2\varphi = \frac{\int_0^{\frac{\pi}{2}} I(\varphi)\cos^2\varphi\sin^2\varphi\, d\varphi}{\int_0^{\frac{\pi}{2}} I(\varphi)\sin^2\varphi\, d\varphi}$, where $I(\varphi)$ is the intensity at the azimuthal angle (φ). Fourier transform infrared (FTIR) spectra were recorded using a Bruker Tensor 27 FTIR instrument. The samples were ground into fine powder, mixed with KBr and pressed into transparent pellets before measurement. To collect data with the sample positioned at different angles to the incident beam, a wider belt sample was prepared by flowing GO colloid through a wide channel having a height equal to the diameter of the nozzle (height 160 μm; width 3 mm). To unequivocally differentiate the bridging C(=O)–NH

group signal from the O–H and C–O–C signals on the graphene sheet surface, partially reduced samples with traces of O–H and C–O–C were analyzed. The belt sample was directly fixed on a Brewster angle holder to accurately control the angle between incident light and normal direction of the sample. Solid state ^{13}C magic-angle spinning (MAS) nuclear magnetic resonance (NMR) spectroscopy was carried out on a Bruker AVANCEIII HD/WB-400 NMR instrument equipped with 3.2 mm H/F/X probe. ^{13}C cross polarization (CP) MAS spectra were recorded with MAS frequency of 10 kHz and recycle delay of 5 s. X-ray photoelectron spectroscopy (XPS) measurements of the freeze-dried GO powder and cut fibers were conducted on an EscaLab 250Xi (Thermo Fisher) using a monochromatic Al-Kα X-ray source. Raman spectra were collected using an HR Evolution (Horiba Jobin Yvon) Raman microscope under 532 nm laser excitation.

Supplementary Figure 1-4. Characterization of GO sheets.

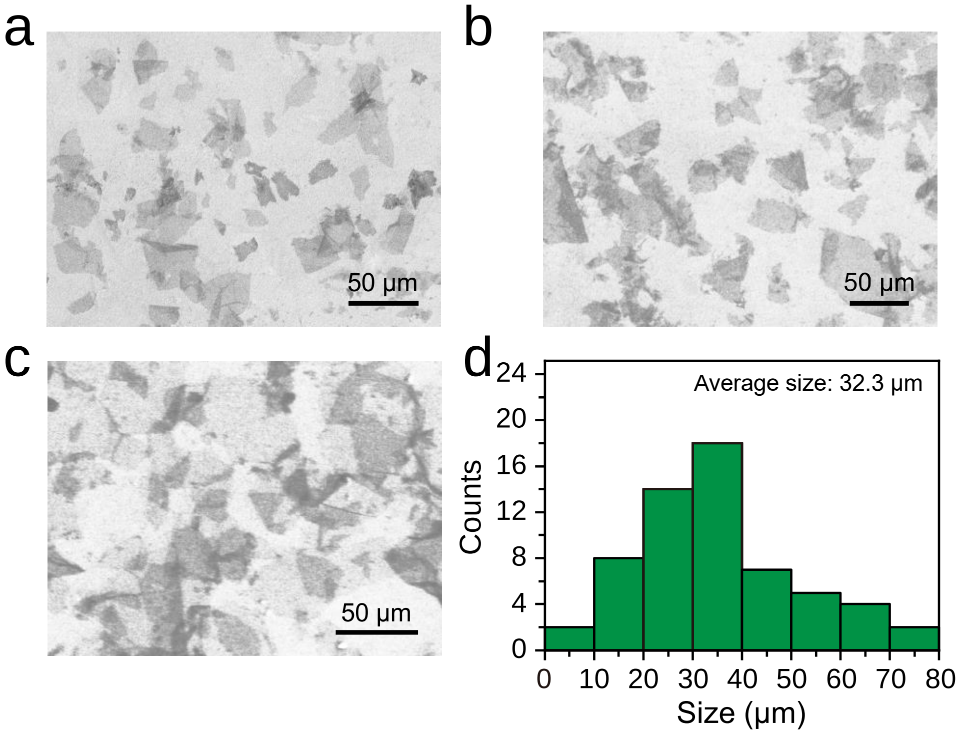

Supplementary Figure 1. SEM characterization of GO sheets.

(a-c) Representative SEM images of GO sheets on FTO substrates. (d) The corresponding histogram of GO sheet lateral size distribution. The sizes of the GO sheets were mostly in the range of 10–70 μm.

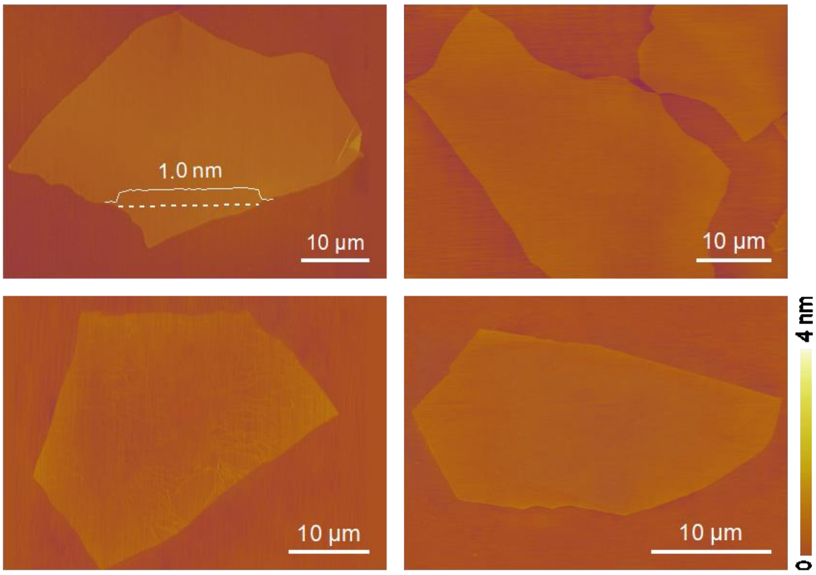

Supplementary Figure 2. Representative tapping-mode AFM images of GO sheets. AFM images of GO sheets deposited on mica substrates displaying typical sheet morphology. Inset: the corresponding height profile. The sheet thickness was approximately 1.0 nm.

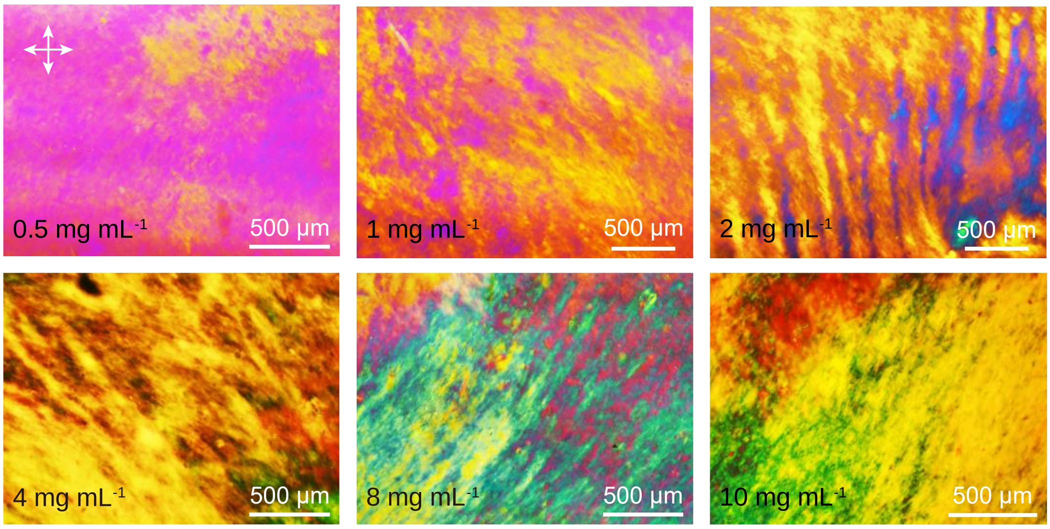

Supplementary Figure 3. Polarized optical microscopy images of colloidal GO sheets.

The images of colloidal GO at increasing concentrations from 0.5 to 10 mg mL^{-1}. It is seen that at concentrations >1.0 mg mL^{-1} GO sheets organized themselves and formed a liquid crystalline phase with local oriented domains, as indicated by the appearance of birefringence under polarized light.

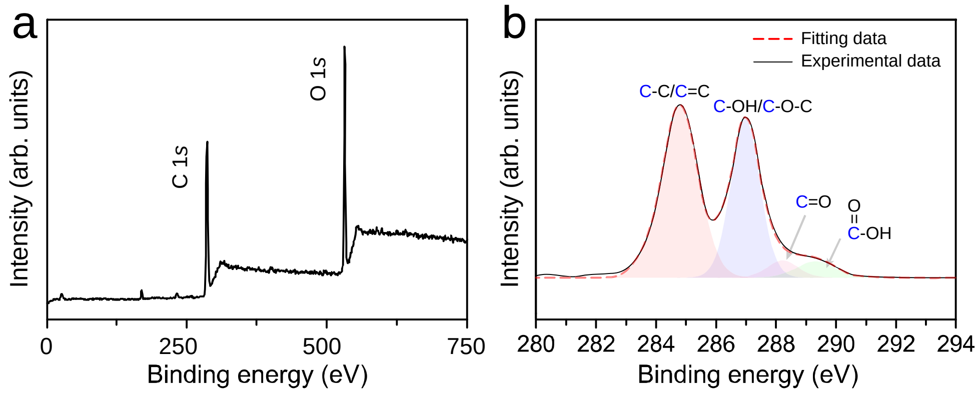

Supplementary Figure 4. XPS characterization for freeze-dried GO (oxidation time 2.25 h).

(a) XPS survey and (b) resolved C 1*s* spectra; experimental data: solid lines, fitting data: short red dashed lines. Besides non-oxygenated C, four different oxygen-containing species are identified, originating from hydroxyl, epoxy, carbonyl, and carboxyl groups.

Supplementary Figure 5-10. Reactivity of oxygen-containing groups on GO with our coagulant, 1,2,4,5-tetraaminobenzene tetrahydrochloride.

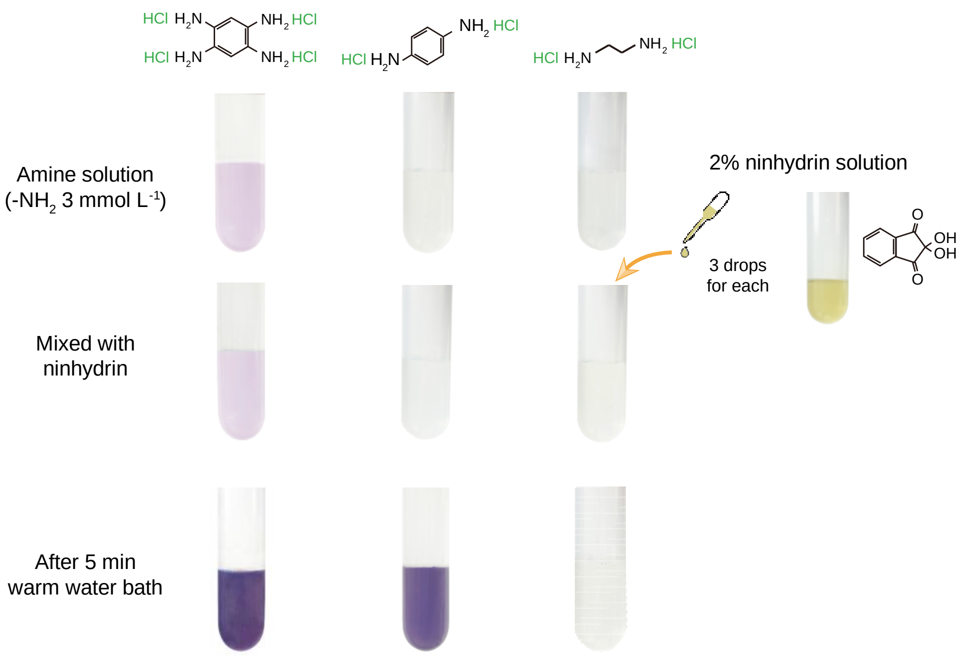

Supplementary Figure 5. Ninhydrin test for the coagulant solution, aqueous 1,2,4,5-tetraaminobenzene tetrahydrochloride. As aqueous 1,2,4,5-tetraaminobenzene tetrahydrochloride itself has a purple color, test with another aromatic amine 1,4-phenylenediamine dihydrochloride was also performed, and compared the results with an aliphatic amine counterpart (ethylenediamine dihydrochloride).

A few drops of ninhydrin solution were added to the test solution, and the tube was kept in a warm water bath for ca. 5 min. The test was performed in an argon-filled box. Both 1,2,4,5 tetraaminobenzene tetrahydrochloride and 1,4-phenylenediamine dihydrochloride gave a positive deep purple color after reacting with ninhydrin, confirming the presence of un-ionized Ar-NH_2 in aromatic amines, even in the presence of acid, which is related to electron delocalization into the benzene ring. In the presence of carboxyl groups, the H^+ ions have a greater tendency to protonate the oxygen on the carbonyl group due to the more stabilized resonance structures of the intermediate. This further increases the quantity of un-ionized Ar-NH_2.

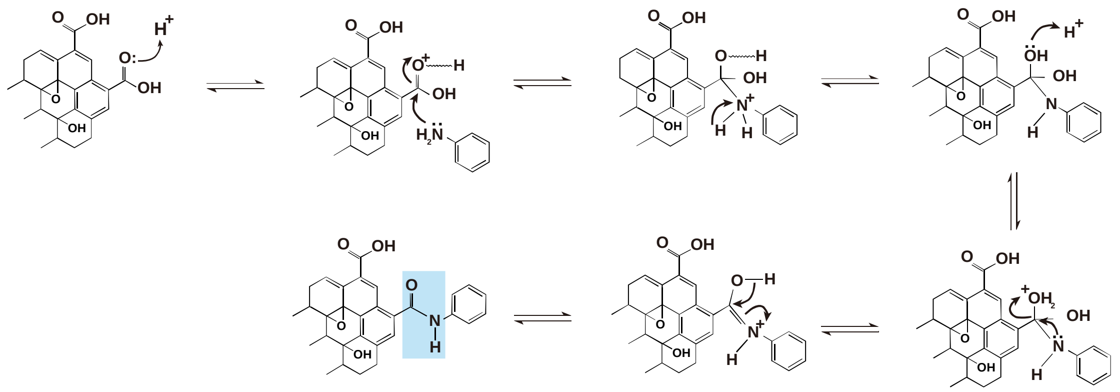

Supplementary Figure 6. Catalytic mechanism of the direct amidation reaction of GO in the presence of acid.

Although the amidation reaction is typically done by acyl substitution to obtain a better leaving group (due to the competing acid-base or electrostatic reaction into a salt), this procedure is tedious and quite complex in practice. Hence, alternative ways to drive direct amidation between carboxyl and amine groups have been studied. For example, it has been reported that GO enhances the electrophilicity of carboxyl groups via hydrogen-bonding;[2] the involvement of an acid also activates the carboxyl group by protonating the carbonyl O that makes it more susceptible to nucleophilic attack.[3] In our case, the combined effect of hydrogen bonding and protonation of the carbonyl O, triggered the direct nucleophilic attack of amines to the carboxyl on GO sheets. The resonance structures involving the graphene-amide-benzene system lead to enhanced stability of the amide and the reaction equilibrium will shift to some extent towards amide formation.

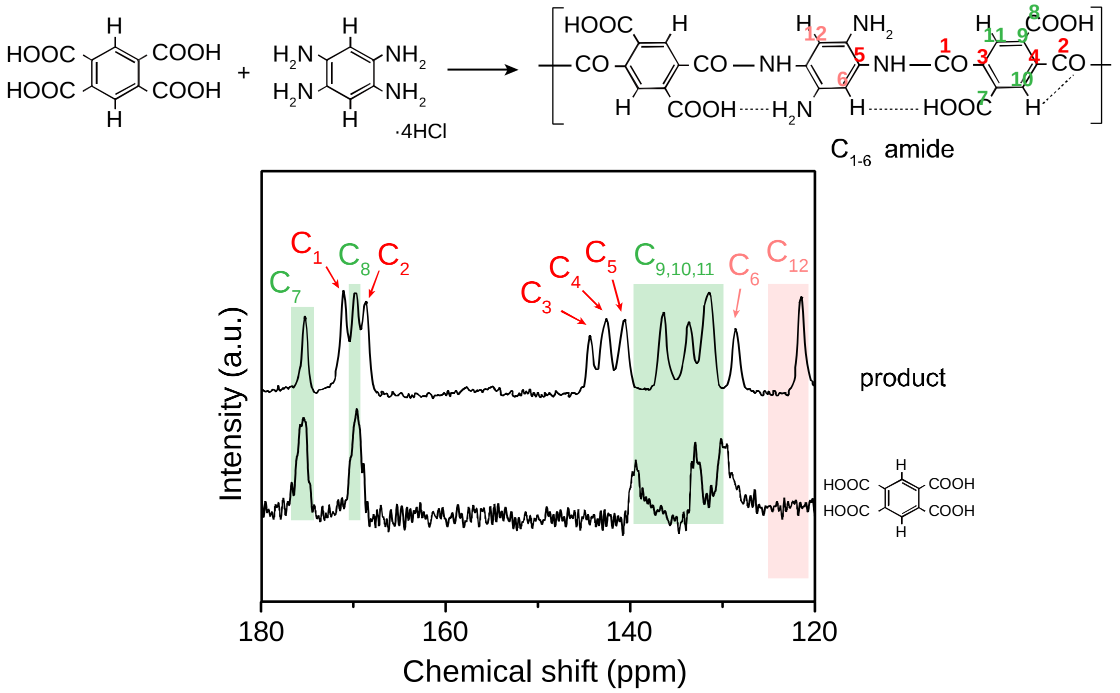

Supplementary Figure 7. ^{13}C NMR for the solid obtained for the model reaction to demonstrate direct amide formation at ambient conditions. The spectra for the reactants are also shown for comparison.

The model reaction of aqueous 1,2,4,5-benzenetetracarboxylic acid 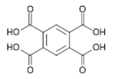 with excess of the selected coagulant of 1,2,4,5-tetraaminobenzene tetrahydrochloride was studied. Aqueous solutions of the two were mixed at room temperature without the addition of catalyst/dehydrator whereupon precipitation was observed immediately after mixing. Clear signals related to amide were observed for the reacted solid, $C_{1\text{-}5}$ in ^{13}C NMR spectrum, pointing to the formation of amide, which confirms that on-site amidation reaction between the carboxyl on graphene and the selected amine is possible.

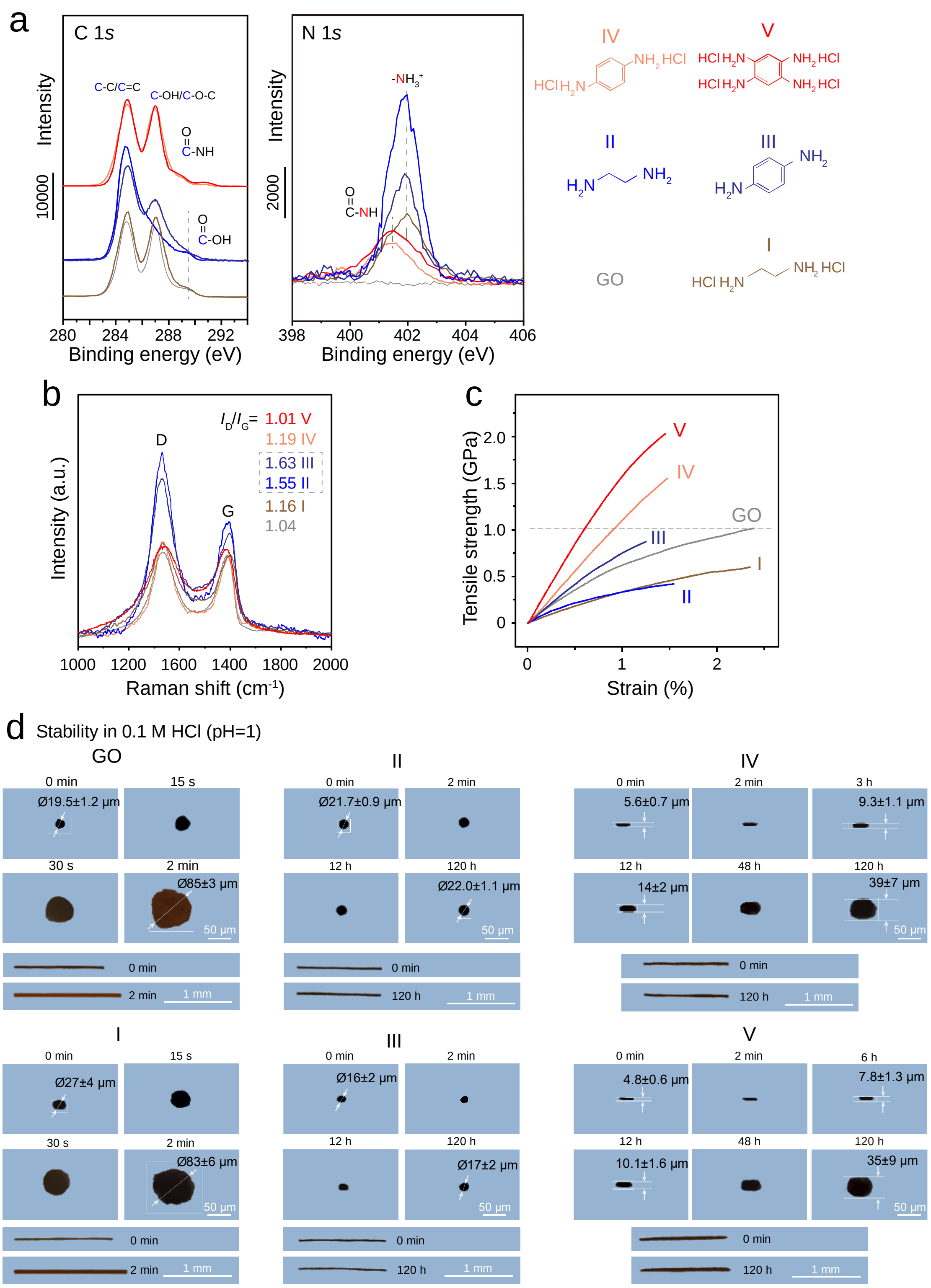

Supplementary Figure 8. Experimental data for fibers coagulated in the different amines to explain the unique chemistry in our system.

(a) XPS, (b) Raman data, (c) stress-strain curves, and (d) stability in HCl. Experimental conditions were the same for all the amines and the amino group concentration was 0.02 M in all cases. Aqueous HCl was selected because water can disrupt the π-π

interaction due to the hydrophilic surface of GO, and acid can break the ion bridge formed by electrostatic interactions. Error bars were estimated from statistical analysis of measurements on 15 locations on each sample.

As discussed in detail below, we used different coagulants classified as, aliphatic amine in the presence of an acid (I), amine in the absence of acid (II and III), and aromatic amine in the presence of an acid (IV and V); the reactions with these coagulants of different type are quite different. The reaction with I is mainly through electrostatic interaction, but with II/III deoxygenation along with some ammonium ion intercalation takes place. The reactions with both I and II/III occur on all the negative charges on GO, resulting in a circular shape of the as-coagulated fibers. In contrast, the relatively selective reaction with groups at GO edge with coagulants IV/V triggers the formation of an unusual belt shape. These different reactions lead to large differences in the bonding between sheets, as demonstrated by their difference in stability in the presence of HCl. Swelling in all directions occurs for I, a strong resistance to swelling and negligible change of shape for II/III, and anisotropic swelling limited to the stacking directions for IV/V.

- Ethylenediamine dihydrochloride

Ethylenediamine, being an aliphatic amine, is easily protonated into the ammonium ion, especially in the presence of an acid, resulting in a weakly acidic pH of 5.5 and a nearly complete protonation (as estimated from p*K*a and pH). The protonated ammonium ions lose their nucleophilic character and cannot form the amide due to the nonavailability of the lone pair of electrons on the N atom. Thus, we attribute coagulation action of ethylenediamine mainly to electrostatic interactions.

Consistent with the above reasoning, there was almost no change in XPS C 1*s* and Raman spectra after coagulation; the N 1*s* XPS signal was seen at a slightly higher energy; the fiber had poor mechanical property and it readily swelled and dissolved in strong acid.

- Ethylenediamine and 1,4-phenylenediamine, examples of diamines used in references

In the absence of acid, the amines also protonate in water to some degree, giving a basic pH 11.2 for ethylenediamine and pH 9.0 for the aromatic amine of 1,4-

phenylenediamine. According to previous reports, treating GO with a base, for example, NaOH, can annihilate oxygen groups.[4] There are also reports where basic amines were used to remove the epoxy and hydroxyl groups from GO. As a result, the coagulation in these cases could be principally due to deoxygenation, along with some ammonium ion intercalation. An obvious decrease in OH/C-O-C signal was observed in XPS C 1*s*. It is noted here that this effect was also observed in the previous cross-linking works with simple amines.[5-8]

Previous reports have also mentioned that a very high density of vacancy defects is usually produced accompanying such oxygen annihilation,[9] due to which the composite membranes produced by this method were reported mainly for separation applications. We also confirmed the increase in vacancy defects from the clear increase in D band in Raman spectra. Consequently, the mechanical strength of the fibers decreased from ~1000 for neat GO to ~405 and 800 MPa for ethylenediamine and 1,4-phenylenediamine, respectively.

These two fibers show strong resistance to swelling, with no obvious change even after being immersed in 0.1 M HCl for >5 days. The resistance to swelling and almost negligible change of shape as well as the circular shape, all suggest high degree of cross-linking with no specific preferential direction. The deoxygenation and the resultant water expelling also contribute to some degree to the resistance to swelling in aqueous HCl.

-1,4-phenylenediamine dihydrochloride and 1,2,4,5-tetraaminobenzene tetrahydrochloride

In these two amines, protonation is not complete even though in the presence of acid, giving a strongly acidic solution. This is due to the strong delocalization of the N lone pair with the benzene ring (the presence of remaining free $-NH_2$ confirmed by NMR in Figure S5). Thus, this coagulant system uniquely includes H^+, $-NH_2$, NH_3^+, very different from the previous cases described above. The strongly acidic media can, to a large degree filter out the electrostatic interaction and other side reactions such as oxygen annihilation reaction, while favoring amide formation reaction between the carboxyl groups and the amino groups.

The obvious shift of carboxyl signal and the almost no change in OH/C-O-C signal of these two systems suggest that the bond formed in this case should be mostly the transformation of carboxyl to covalent amide. The differences in N 1*s* chemical shifts

are generally small, but signals for these two occurring at lower binding energies confirmed the formation of covalent amide. Raman spectra show almost no change in D-band, confirming that the in-plane structure is maintained. Although the amount of included N could be low, it leads to a significant increase in mechanical properties of the coagulated GO fiber (strength of ~1.6 GPa for 1,4-phenylenediamine dihydrochloride and 2.0 GPa for 1,2,4,5-tetraaminobenzene tetrahydrochloride). This, we believe, should be due to a synergistic effect between maintenance of in-plane structure, lateral bridging, and enhanced π-π interactions.

The stability test shows swelling, but limited to the stacking direction and requiring much longer time. The stability in the axial direction should be largely due to high degree of chemical bridging; the slow swelling in the stacking direction may suggest improved π-π interaction resulted from conjugated bridging of sheet edge or some low-degree chemical cross-linking. The unique anisotropic swelling behavior confirms the selective chemical cross-linking in our strategy.

The almost no change of in-plane structure and the selective swelling in the stacking direction meantime suggest that ester formation between hydroxyl groups on GO with the carboxyl group, if any, should be minor in this case.

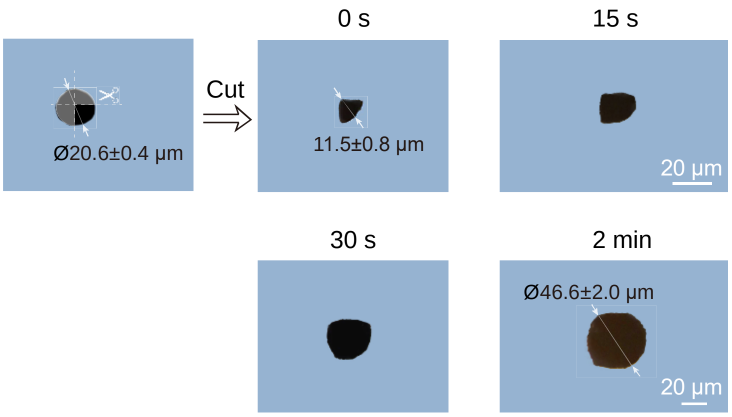

Supplementary Figure 9. Possible effect of shape on swelling behavior.
Stabiliy of the fiber with coaguant I under the same condition as in Figure S8, except that the circular shape was cut from the top and left-side into a flat piece before adding HCl to initiate swelling. Error bars were estimated from statistical analysis of measurements on 15 locations on each sample. We also tried to cut the solvent-exchanged GO sample in a similar way, but this was not possible because the sample easily broke into several small parts, probably due to the weak binding of sheets.

With swelling and time, the flat morphology gradually evolved into a round shape, largely to minimize surface energy. This result shows that the original shape of the fiber does not play a determining role in the swelling behavior, which is instead mainly determined by the bonds that assembles the sheets. In the absence of a chemical bond to restrict swelling (I), the fiber will evolve with time into a circular shape so as to minimize its surface energy; if swelling is restricted in all directions (II and III), no swelling would be observed. If the restriction is present only in certain direction, then anisotropic swelling occurs.

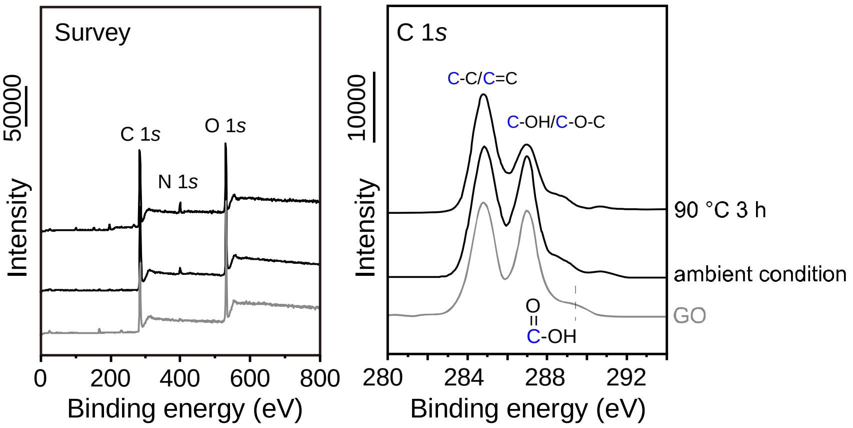

Supplementary Figure 10. Difference in reactivities of the groups on GO.

XPS survey and C 1*s* spectra of the as-obtained fiber sample at ambient condition compared with that heating at 90 °C for 3 h and pristine GO.

For the as-obtained sample, a shift of the carboxylic acid peak to lower energy was observed; the C-OH/C-O-C signals did not change much in the absence of heating, implying that at room temperature it is mainly the carboxyl group that reacts with the amine.

Supplementary Figure 11-12. The effect of linking.

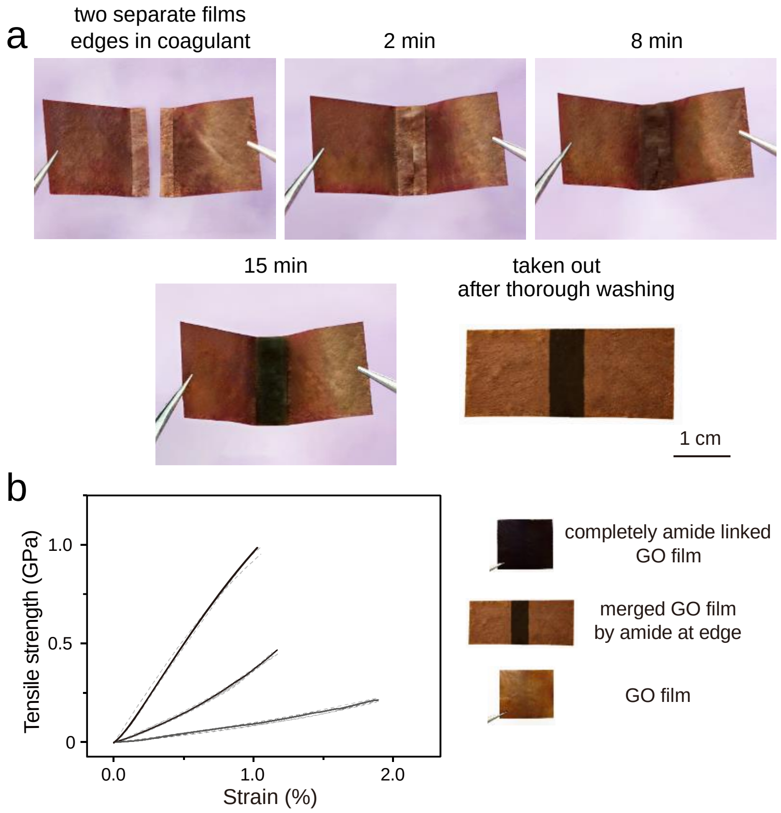

Supplementary Figure 11. Lateral bridging of two separate films and the amidation solidifying process by immersing the edges in aqueous 1,2,4,5-tetraaminobenzene tetrahydrochloride.

(a) Digital images, (b) mechanical property of the merged GO film by amide bridges at the edge, compared with the original GO film, and the completely amide-bridged GO film. The films cannot be connected with only water, and the parts immersed beneath tend to soften and dissolve.

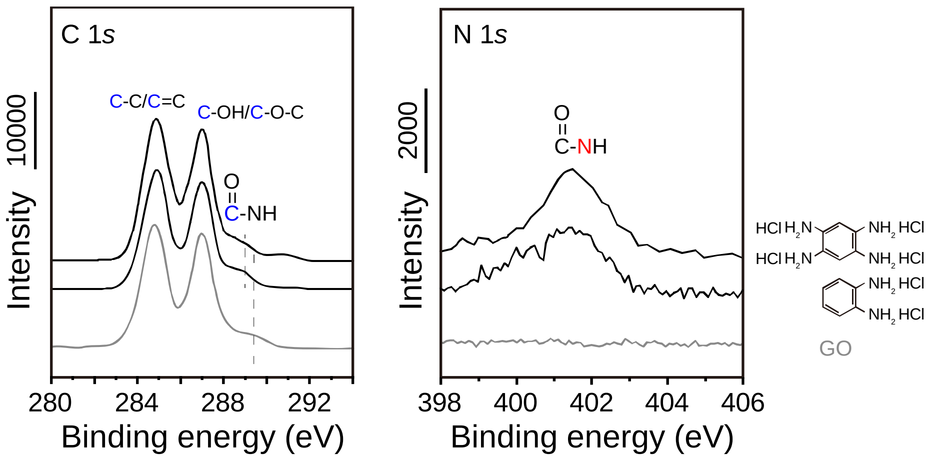

Supplementary Figure 12. Confirmation of the presence of linking by the groups at para positions.

XPS of the filtrated colloid of GO injected into aqueous amine of 1,2-diaminobenzene hydrochloride (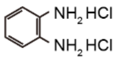), with amino groups on the same side of the benzene ring, in comparison with 1,2,4,5-tetraaminobenzene tetrahydrochloride (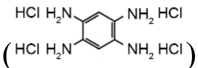). No fiber was formed when using 1,2-diaminobenzene hydrochloride and the resultant colloid was characterized. The formation of the same type of bond, whether the amino groups were on the same side or on opposite sides of the benzene ring, and the absence of fiber formation in the former strongly support the presence and effectiveness of linking by the groups at para positions in our fiber system.

Supplementary Figure 13-15. Characterization of control fiber prepared by solvent exchange protocol.

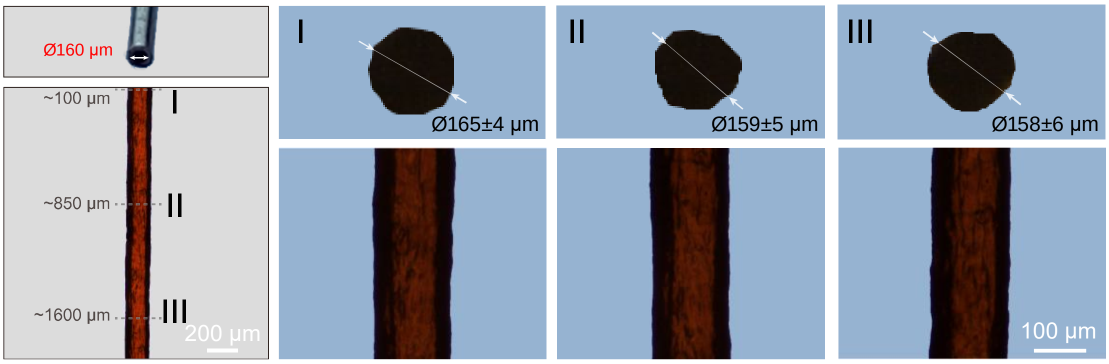

Supplementary Figure 13. Morphology characterization of control GO fiber from a circular nozzle.

Optical microscopy images of cross-sections of freshly coagulated GO fiber by the most commonly used solvent exchange protocol. The fiber replicates the round geometry of nozzle. Error bars were estimated from statistical analysis of measurements on 15 locations on each sample. The labels I, II, III in the left panel indicate the locations of cross-sectional observation and the numbers given alongside are the distances from the nozzle exit.

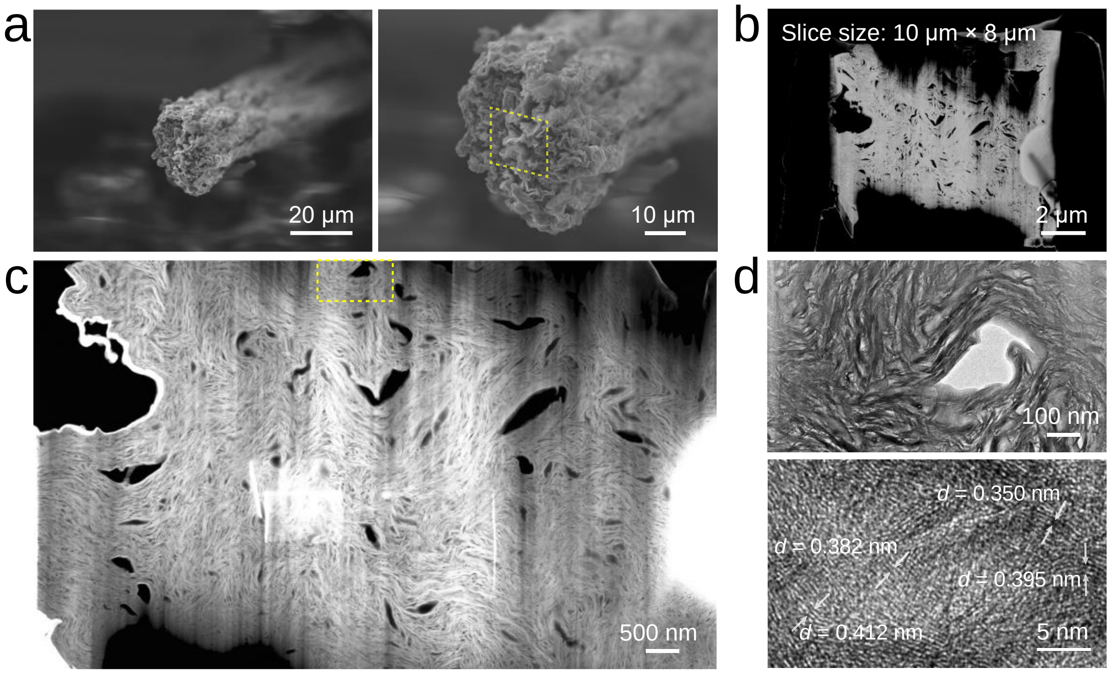

Supplementary Figure 14. Cross-sectional images of the control graphene fiber coagulated by solvent exchange.

(a) SEM images, (b–c) STEM-in-SEM images, and (d) TEM images. The circular geometry of the nozzle and the random sheet orientation in the starting aqueous dispersion are maintained in the coagulated fiber.

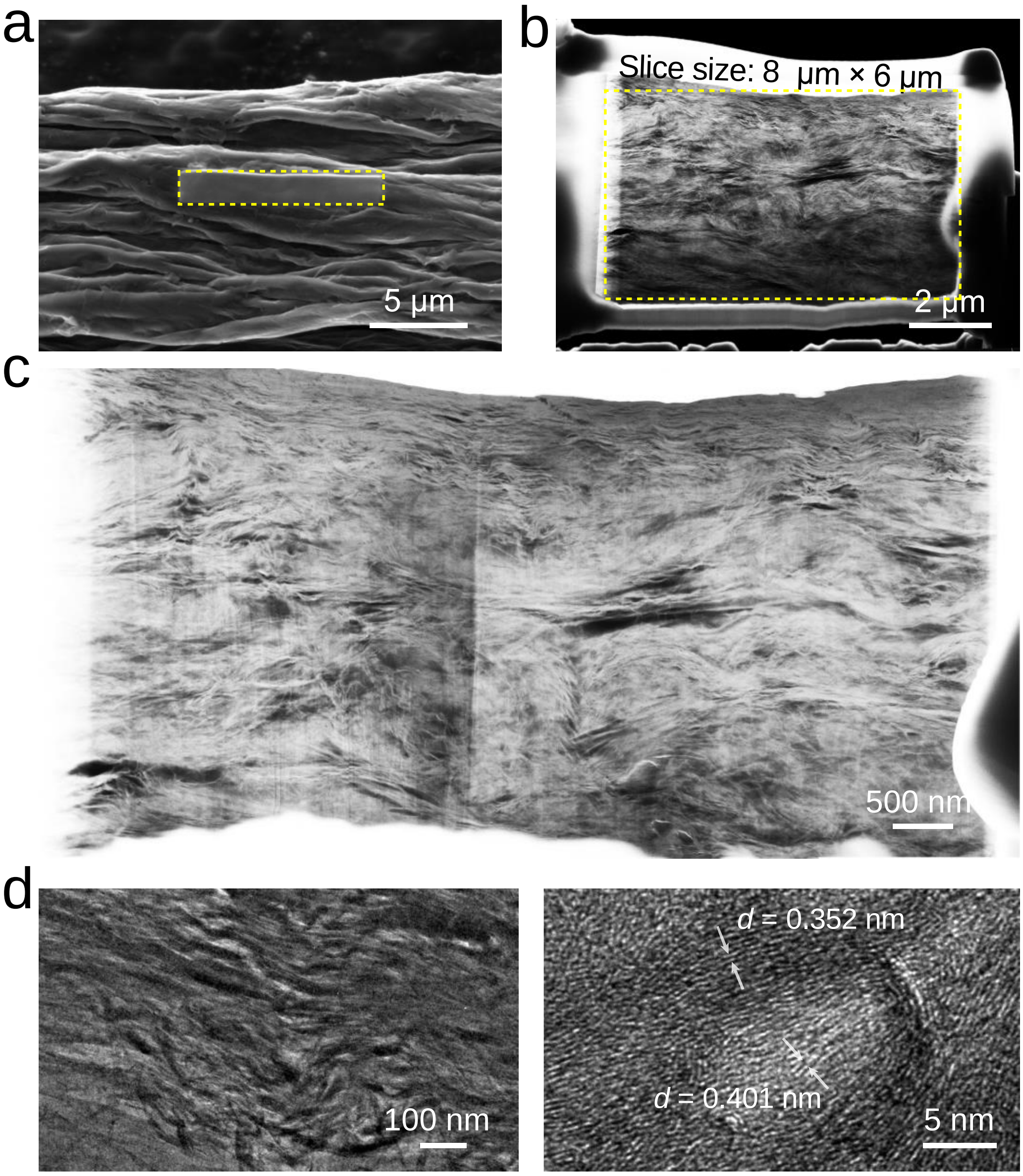

Supplementary Figure 15. Imaging along the longitudinal direction of the control graphene fiber formed by solvent exchange.

(a) SEM image, (b–c) STEM-in-SEM images, and (d) TEM images. Stretching was applied in this direction during drying, because of which some narrow areas of alignment can be found similar to that reported recently for a high-performance fiber,[10] but the sheets are still largely misaligned.

Supplementary Figure 16-24. The solidifying process and characterization of GO fiber formed by amide bridging.

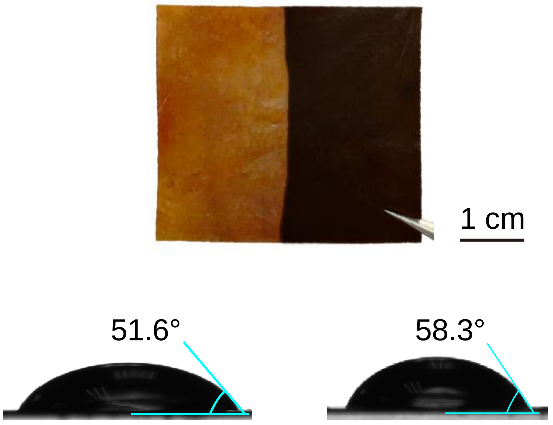

Supplementary Figure 16. Examination of possible hydrophilicity change.

Hydrophilicity measurement of a film half of which was subjected to amidation using the aromatic amine coagulant (1,2,4,5-tetraaminobenzene tetrahydrochloride); the contact angle is changed only slightly.

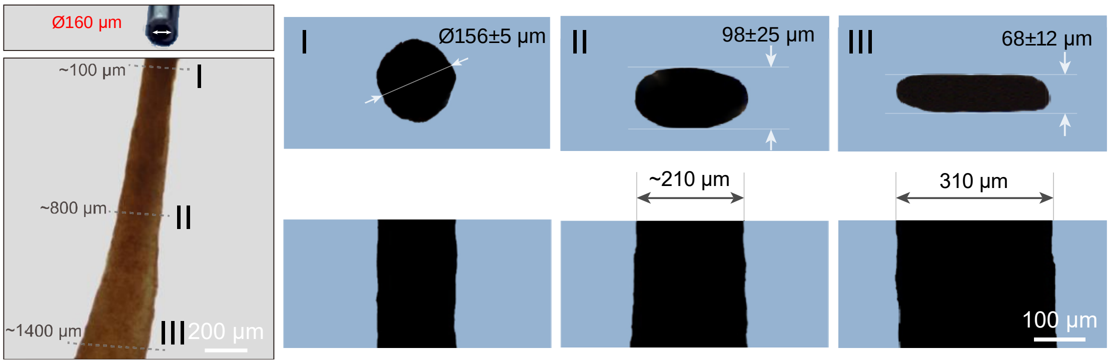

Supplementary Figure 17. Tracking the solidification and flattening process. Optical microscopy images of cross-sections of the freshly coagulated wet GO fiber, showing the flattening into a belt-shape immediately after the colloid is extruded out of the nozzle. Error bars were estimated from statistical analysis of measurements on 15 locations on each sample. The I, II, III in the left panel indicates the locations for cross-sectional observation and the numbers next to them are the distances from the nozzle exit.

According to the distance of the fiber from the nozzle and the experimental parameters including rotation rate and the radiating distance of the fiber from the center of the rotating disk, the time to achieve flattening can be roughly calculated to be ~18 ms.

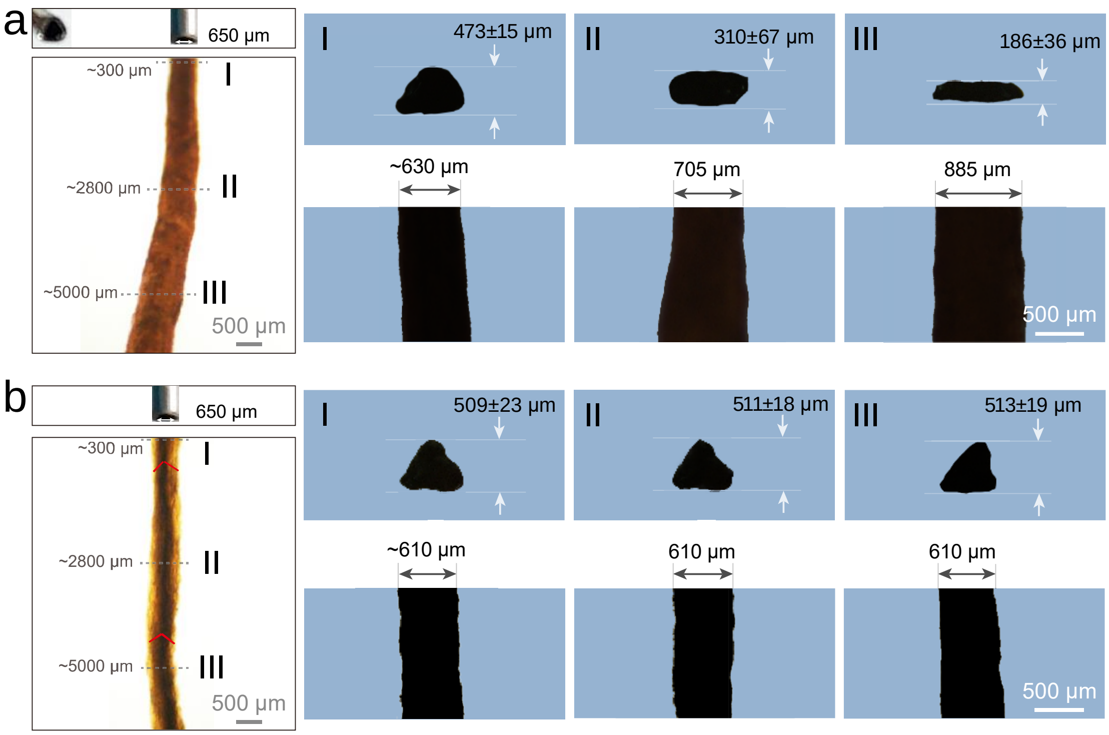

Supplementary Figure 18. Effect of the nozzle shape.

Optical microscopy images of (a) our GO fiber system and (b) the control fiber system solidified by solvent exchange, using a nozzle of triangular shape. Error bars were estimated from statistical analysis of measurements on 15 locations on each sample. The I, II, III in the left panel indicate the locations of the observed cross-sections and the numbers given alongside are the distances from the nozzle exit.

It is clearly seen that the amide fiber still shows a belt shape, while the control system replicates the geometry of the nozzle, giving a triangular morphology.

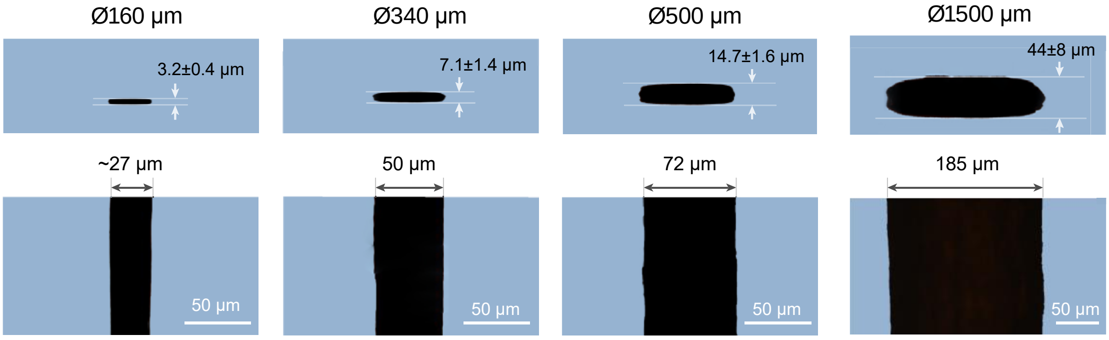

Supplementary Figure 19. Effect of diameter of tubular channels.

Optical microscopy images of dry GO fibers spun with tubular channels of varying diameters (left to right): ⌀=160, 340, 500, and up to the test limit of 1500 μm. Error bars were estimated from statistical analysis of measurements on 15 locations on each sample. For all cases, the fibers show a belt-like morphology.

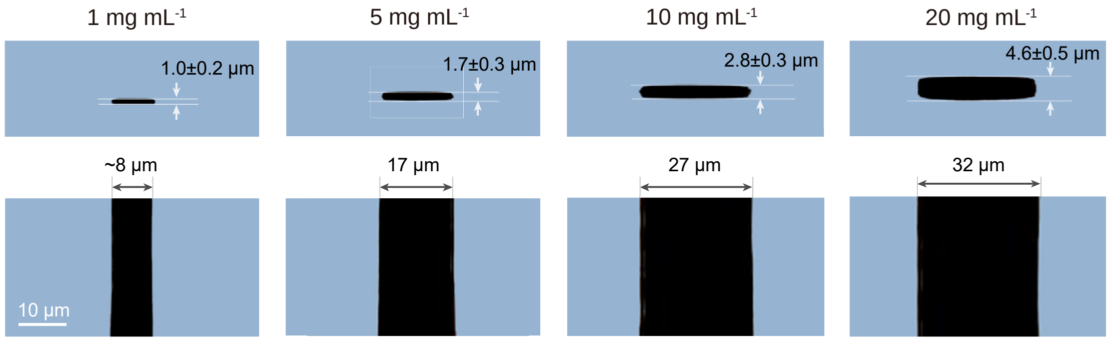

Supplementary Figure 20. Effect of GO concentrations.

Optical microscopy images of dry GO fibers spun with GO concentrations of 1 to 20 mg mL^{-1} through a tubular channel of ⌀=160 μm. Error bars were estimated from statistical analysis of measurements on 15 locations on each sample. The belt thickness varies with GO concentration.

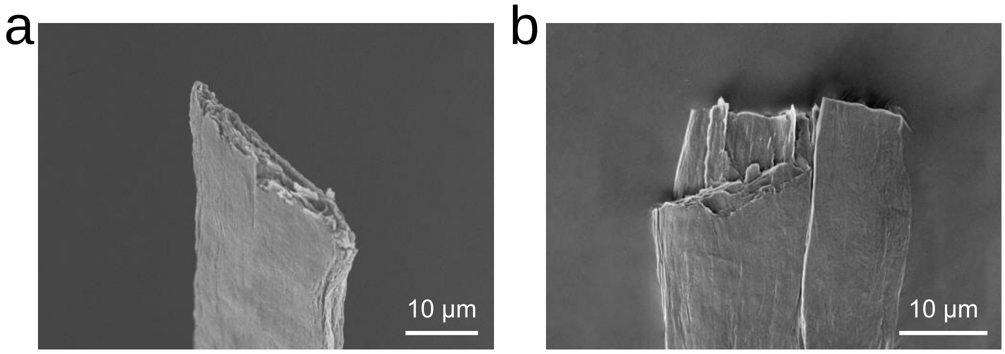

Supplementary Figure 21. SEM characterization of GO belt fibers.

(a) Representative SEM image of a GO belt fiber and (b) the surface peeled with tape, showing the compact structure and good alignment in both the stacking and longitudinal directions.

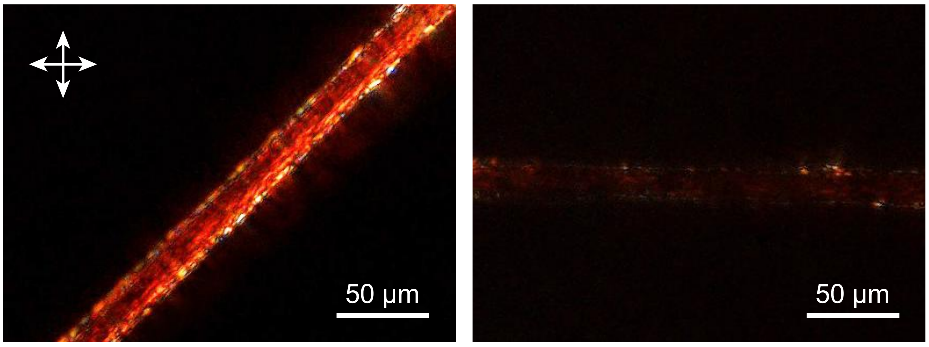

Supplementary Figure 22. Characterization of ordering in fiber by polarized optical microscope.

Polarized optical microscopy images of GO fiber with the fiber axis at 45° and 0° with the direction of polarizer. The bright and uniform birefringence color at 45° and dark appearance at 0° implies high alignment of sheets along the fiber axis.

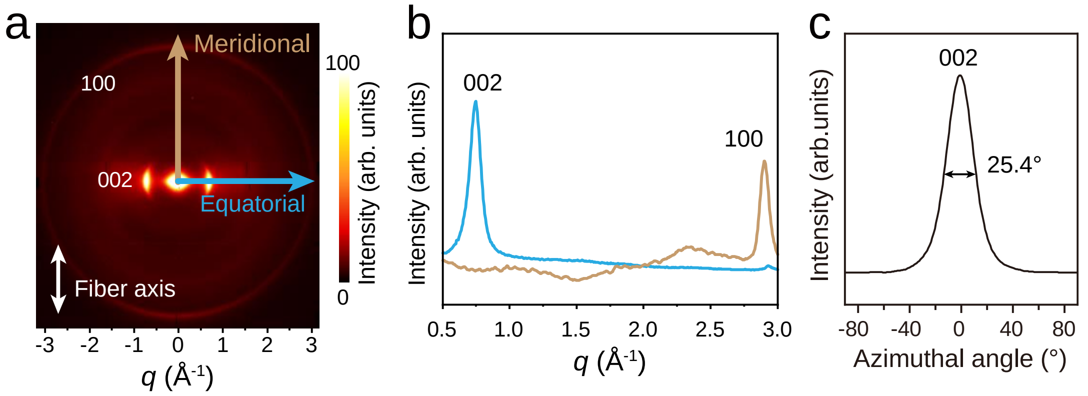

Supplementary Figure 23. WAXS data from representative fibers.

(a) Two-dimensional WAXS pattern of GO fiber, (b) the corresponding radial scan curves integrated in the equatorial and meridional directions, and (c) azimuthal scan profile for the (002) peak. The GO fiber also shows a high orientation degree with orientation angle as small as 25.4°. The estimated orientation factor is 0.856.

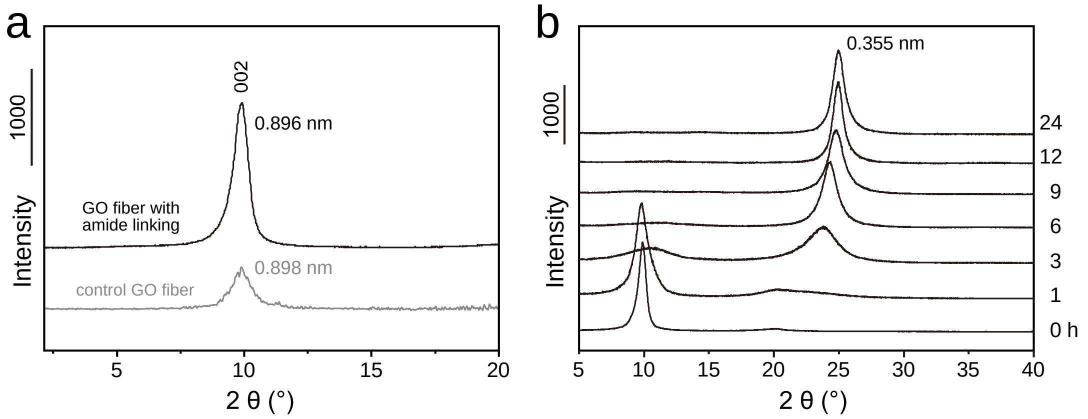

Supplementary Figure 24. XRD data for GO fiber and the fibers in the reduction process.

(a) XRD pattern of GO fiber. The data for the control fiber is also shown for comparison. (b) XRD patterns for fibers reduced by HI for different time periods.

The basal spacing for the GO fiber is calculated to be 0.896 nm, close to that for control fiber of neat GO sheet stacking, indicative of the absence of guest molecules between sheets and selective reaction at edge. The decreased FWHM of the basal reflection as compared to the control fiber indicates improvement in ordered stacking of sheets.

Upon HI reduction by immersing sample in aqueous HI at room temperature, a new basal peak at higher angles is detected, indicating a reduction in sheet-to-sheet spacing likely due to the removal of oxygen functionalities. Complete reduction is observed after ~12 h of reduction. If the reduction treatment is by exposing sample to HI vapor, a gradual shift of the basal peak to higher angles was typically observed.

Supplementary Figure 25-31. Morphological and structural characterization of our amide-connected graphene fiber.

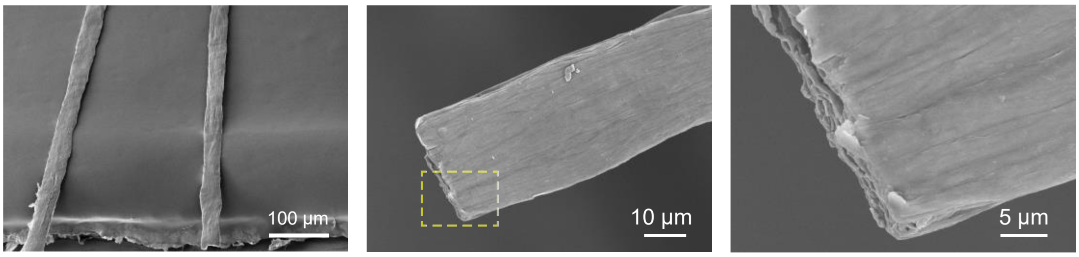

Supplementary Figure 25. SEM morphology characterization of amide-connected graphene fiber.

Representative SEM images of our aromatic amide-connected graphene fiber, showing belt morphology with regular and compact stacking of sheets.

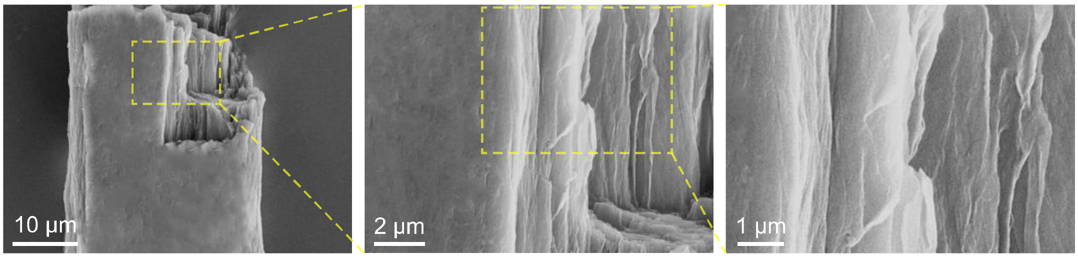

Supplementary Figure 26. SEM characterization of fiber inner structure.

SEM images of example graphene fibers at different magnifications, showing the microstructure along the longitudinal direction. To better visualize the underlying structure, a thin surface layer was peeled off by mechanical exfoliation.

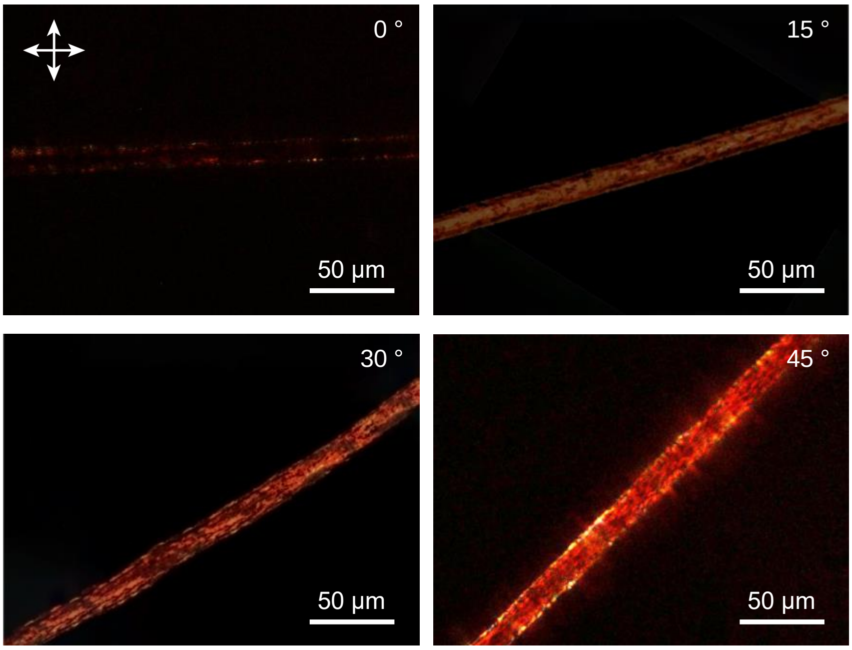

Supplementary Figure 27. Characterization of ordering in fiber by polarized optical microscope.

Polarized optical microscopy images of a graphene fiber with the fiber axis at 0°, 15°, 30°, and 45° with the direction of polarizer. The bright color at 45° and the homogeneous birefringence color along the whole fiber indicate that the sheets are well aligned in the fiber.

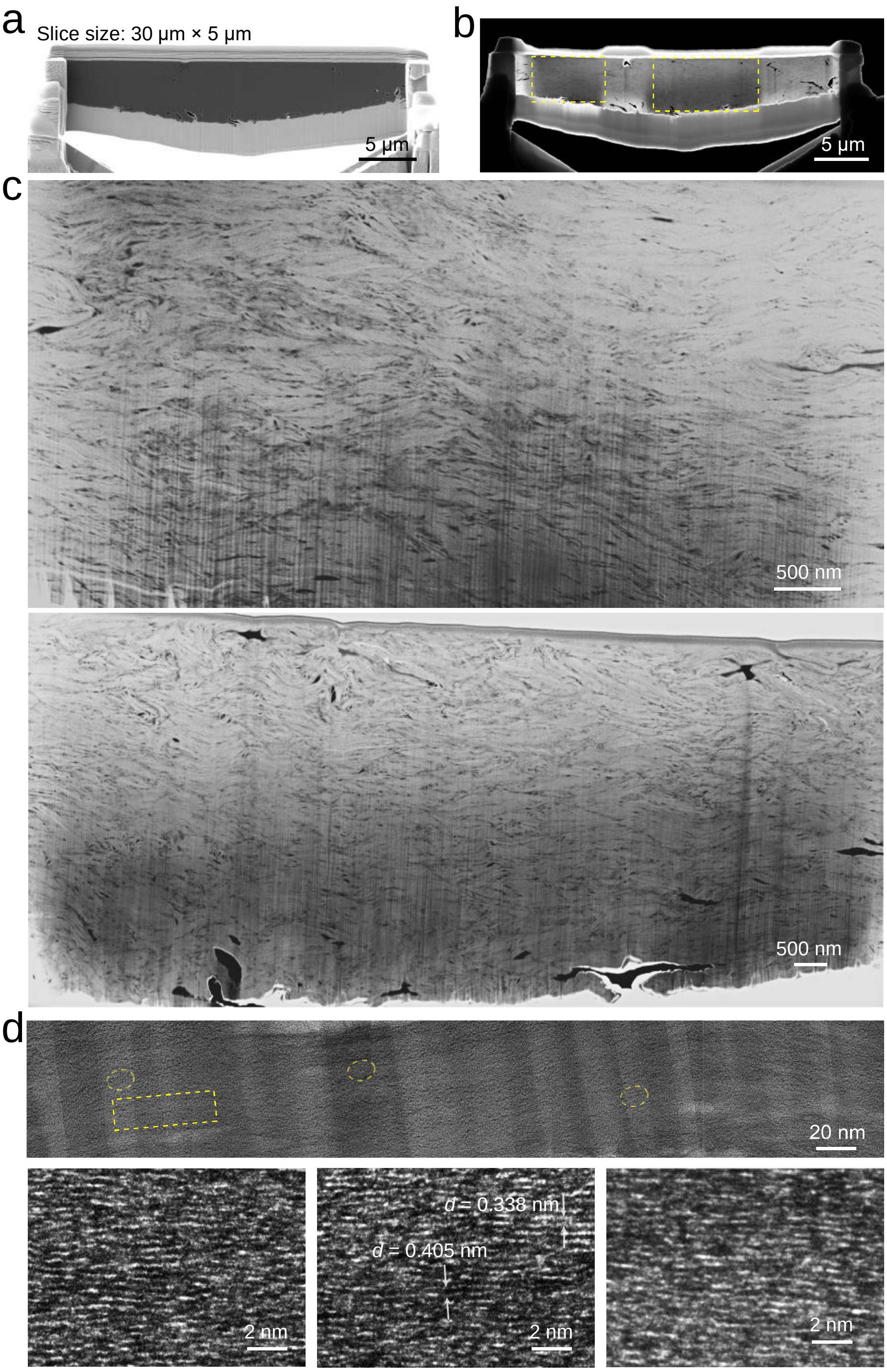

Supplementary Figure 28. Cross-sectional images of the aromatic amide-connected graphene fiber.

(a) SEM image of cross-section cut by FIB, (b) HAADF STEM-in-SEM image of a thin slice of the cross-section sample. The sample needs to be thinned to <100 nm to allow electron penetration, but this 30 μm wide sample was easily bent and even broken and therefore only parts of the sample (marked with a rectangle) were thinned for further

observation. As the cross-section is compact in (a), the holes in (b) are mainly from the thinning process.

(c) STEM-in-SEM images of the thinned area, showing the largely aligned sheets over the whole area, in obvious contrast to the readily observed large curves in the control fiber (Figure S14), and (d) HR-TEM images, displaying the stacked local structure. The rectangle indicates the area for zoom-in HR-TEM view in Figure 2d.

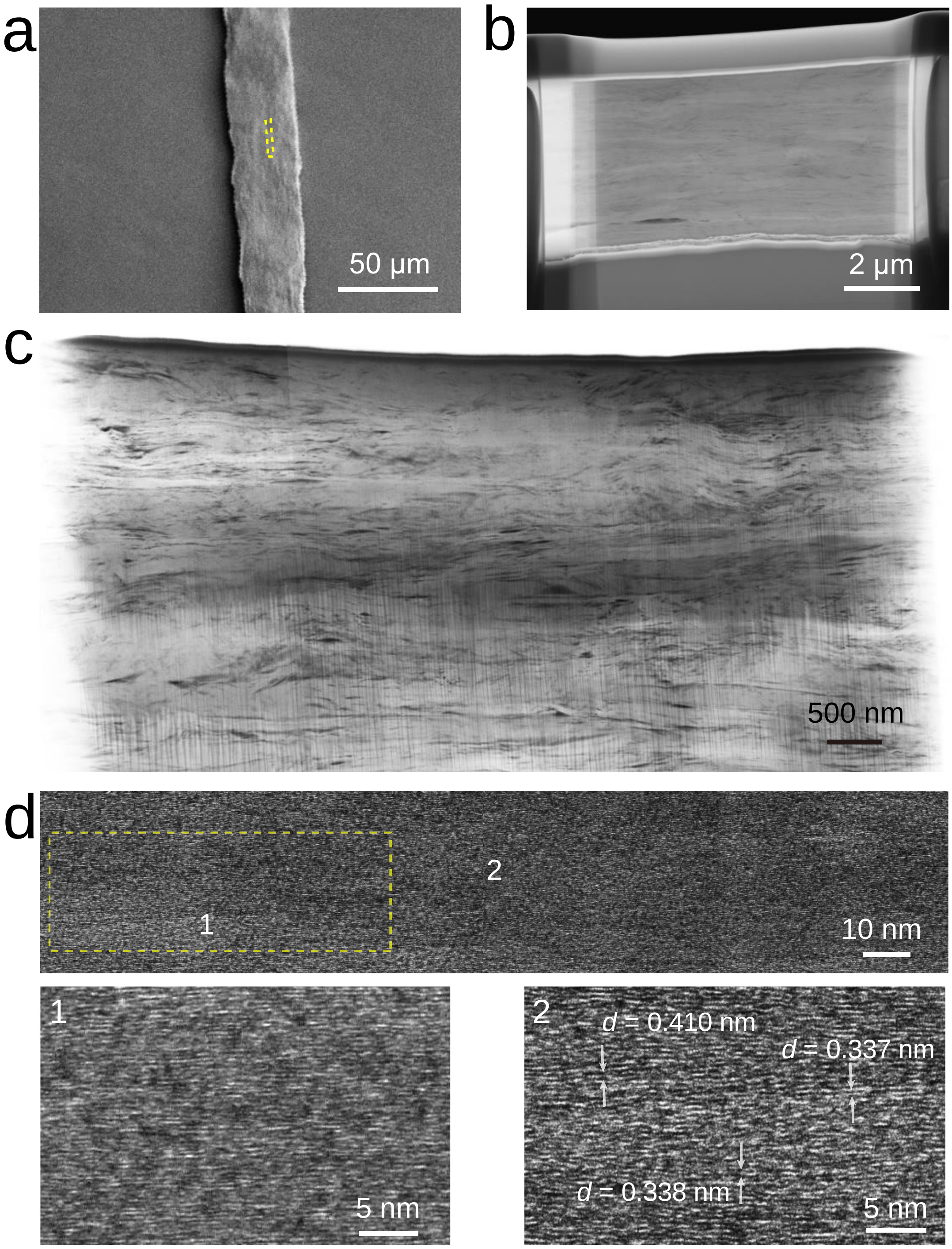

Supplementary Figure 29. Characterization of the amide connected graphene fiber along the axial direction.

(a) SEM image of a fiber; the position of cutting is indicated by the yellow dashed rectangle, (b–c) low- and high-magnification STEM-in-SEM images of the whole section, and (d) typical HR-TEM images showing the well stacked structure. The rectangle indicates the area for zoom-in HR-TEM view in Figure 2e.

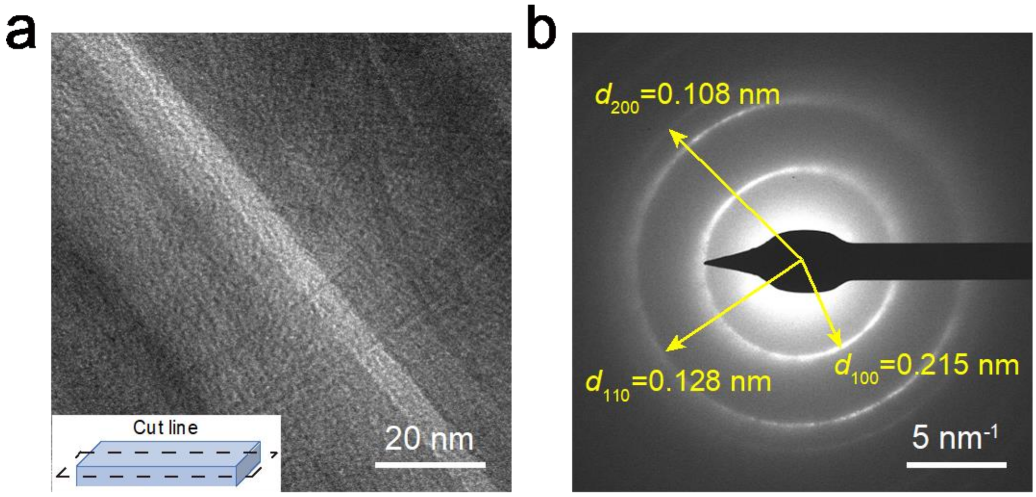

Supplementary Figure 30. TEM characterization for the amide connected graphene fiber.

(a) In-plane-view TEM image and (b) the corresponding SAED pattern.

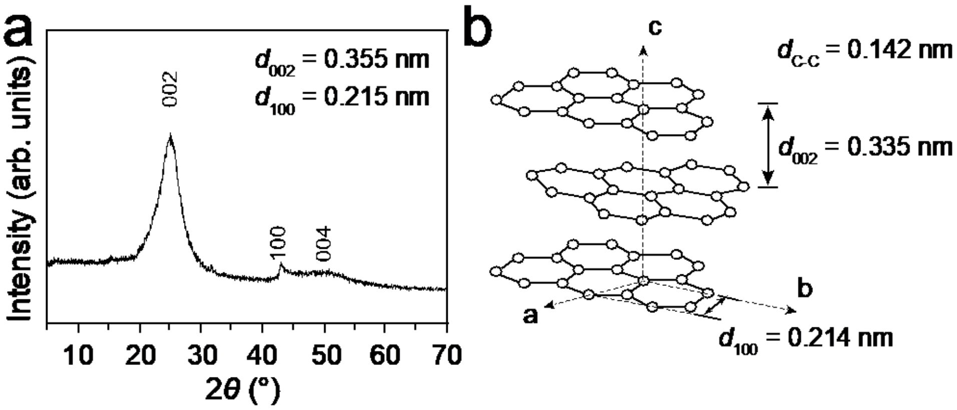

Supplementary Figure 31. XRD characterization for the amide connected graphene fiber.

(a) XRD pattern, and (b) the ideal structure and geometry parameters of graphite. Diffraction peaks corresponding to stacking (002) and in-plane crystallographic order (100) are detected. The measured inter-planar spacing in the stacking direction is 0.355 nm. The slightly larger value than that of ideal graphite seen in XRD and in HR-TEM may originate from some stacking faults. Although the sheets are largely aligned in parallel, the incomplete recovery of the sp^2 carbon network (Figure S42) restricts perfect graphitic stacking.

Supplementary Figure 32-35. Structural characterization of GO and graphene fibers.

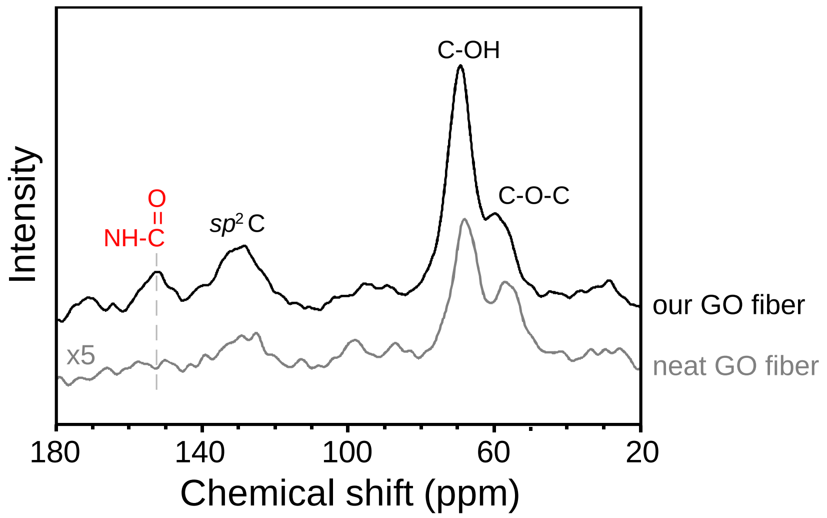

Supplementary Figure 32. NMR data.

Solid-state ^{13}C cross-polarization MAS-NMR spectra of a GO fiber coagulated with the chosen aromatic amine and for the control GO fiber solidified by solvent exchange.

For this measurement, the fiber was ground into powder. Grinding was controlled at minimum level so as to not damage the sample and preserve data quality. It is noted that only resonances from carbons which are very close to relatively immobile protons are seen in the CP spectra of solid-state NMR. The high signal-to-noise ratio in our GO fiber sample spectrum is indicative of grafting of immobile H-containing functions on the GO sheets. Carboxyl group is rarely detected in GO by ^{13}C cross-polarization MAS-NMR; the additional clear signal at chemical shift of ~150 ppm confirms that the immobile H comes from the amide.

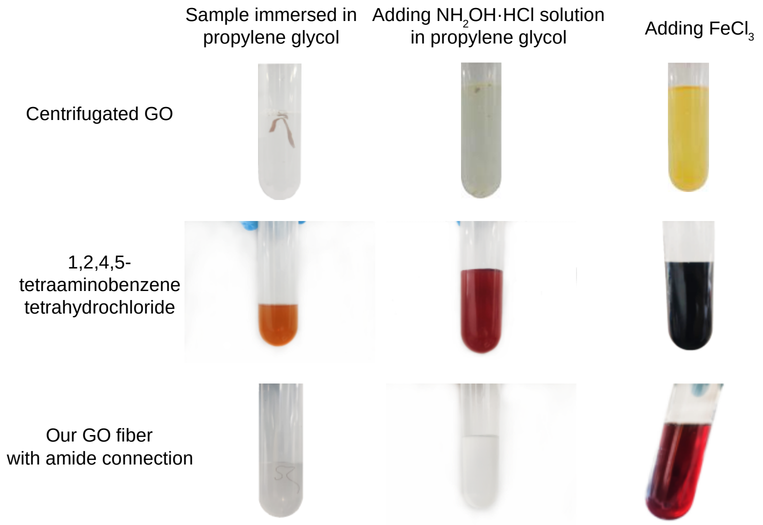

Supplementary Figure 33. Confirmation of amide generation by hydroxamic test. At high temperatures, amides are converted to hydroxamic acids by hydroxylamine,

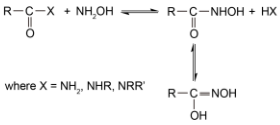

, and hydroxamic acid forms a red complex with iron (III) ion,

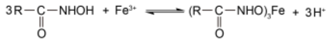

. The ammonium ion does not show such a reaction.

Experimental details: To 10 mL of 1 mol L^{-1} hydroxylamine hydrochloride ($NH_2OH \cdot HCl$) solution in propylene glycol we added 150 mg of the test sample dissolved in minimum amount of propylene glycol. Then, 5 mL of 5 mol L^{-1} potassium hydroxide (KOH) in propylene glycol was added and the mixture was boiled for 2 min. After cooling, 1 to 2.5 mL of 10% iron (III) chloride ($FeCl_3$) solution was added to the mixture. A red color is a positive test for amide groups.

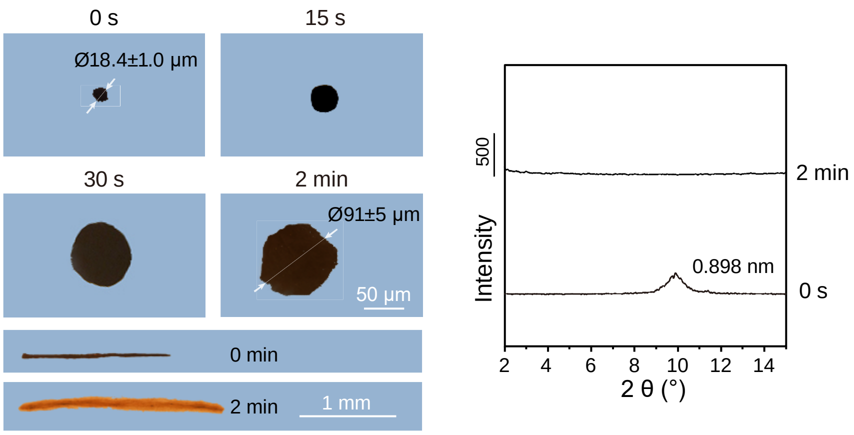

Supplementary Figure 34. Stability of control GO fiber in NMP.

Optical images showing swelling in all directions of the control fiber in NMP; XRD patterns of the original fiber and the fiber after complete swelling are shown on the right. Error bars were estimated from statistical analysis of measurements on 15 locations on each sample.

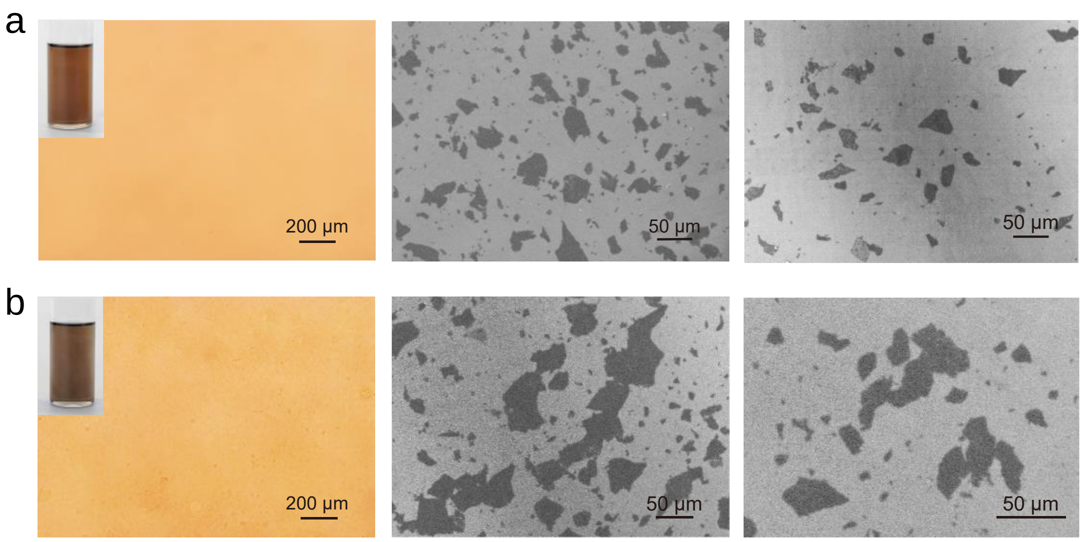

Supplementary Figure 35. Characterizations of sheets before and after linking. The bridged sheets were obtained by chemically exfoliating the swollen fiber in NMP. (a) Digital and optical microcopy images, and SEM images of pristine GO. (b) The data for the colloid delaminated from swollen fiber, occasionally showing broken bridged sheets. The sample for SEM imaging was prepared by dropping diluted colloid on a porous AAO substrate to ensure sheets in dispersed state and avoid possible sheet aggregation in drying.

Experimental details: 5–50 μm size GO sheets were sieved by centrifugation at 2500–7000 rpm (678–5314 $\times g$). The spun fiber was cut into very short segments and immersed in NMP for ~3 d, by which an almost complete swelling was achieved. The sample was then subjected to mechanical shaking for 6 h to yield a fully dispersed colloid. The coagulated fiber can be fully delaminated and almost no fragmented debris were seen under optical microscope, confirming that the interlayer force is mostly π-π interaction.

Supplementary Figure 36-42. Optimization of experimental conditions to achieve best mechanical performance.

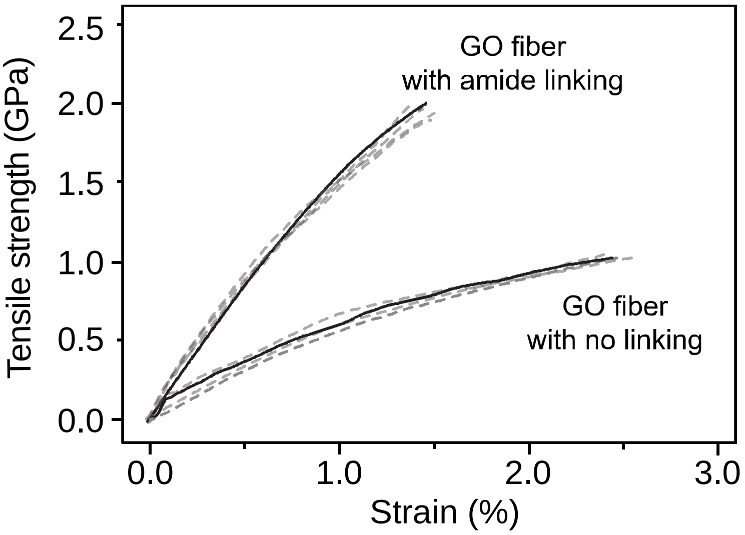

Supplementary Figure 36. Mechanical property test of GO fibers.

Typical stress-strain curves for our GO fibers with amide connection. The data for samples with no connection are also shown for comparison.

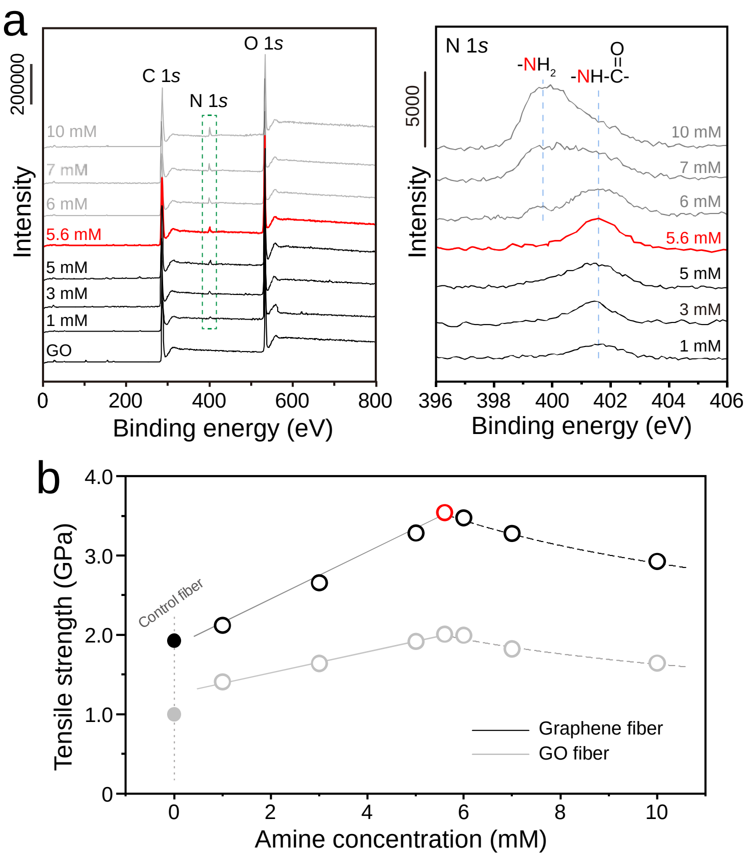

Supplementary Figure 37. Mechanical performance comparison of fibers solidified with different concentrations of the coagulant.

(a) XPS survey and N 1*s* spectra of GO fibers and (b) mechanical performance as a function of the amine concentration.

In the low concentration range, the increase in coagulant concentration increases the intensity of the N signal in the survey spectrum. In addition, the presence of the amide signal in N 1*s* spectrum with little remaining –NH_2, implies increase in amide bridging. Further increase in amine concentration does not bring additional increase in the signal related to amide, but signals corresponding to –NH_2 are greatly intensified. As all the samples were thoroughly washed, this suggests an increase in remaining unreacted –NH_2 at excess coagulant concentration. The presence of significant amount of remaining –NH_2 would result in incomplete edge-to-edge connection and the interactions among linkers may become dominant, yielding inferior performance.

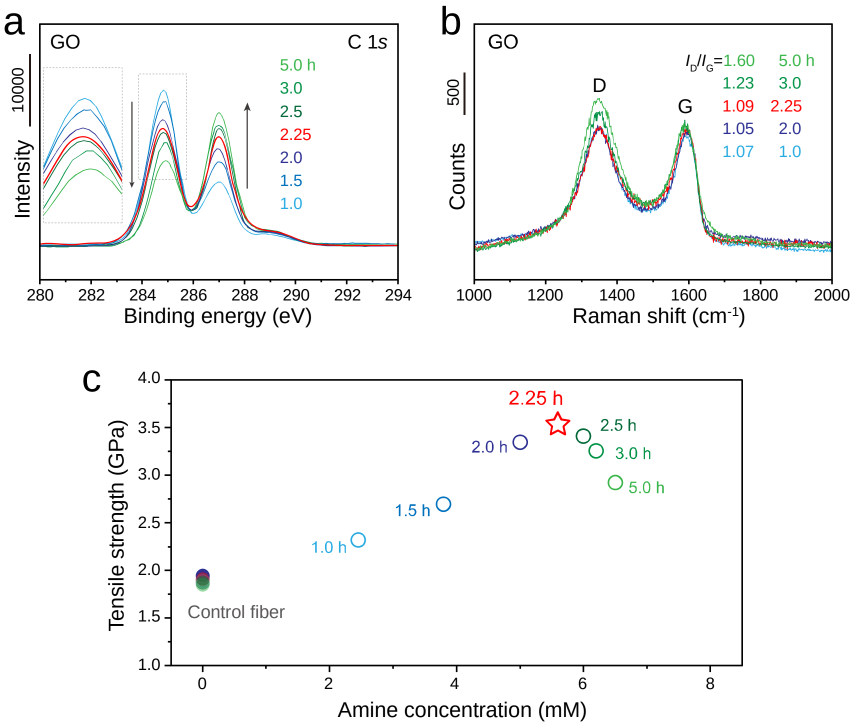

Supplementary Figure 38. Mechanical performance comparison of fibers prepared with GO of differing oxidation degree.

(a) XPS C 1*s* and (b) Raman spectra of GO sheets prepared with different oxidation times. It is noted that coagulant concentration was optimized for each GO with different oxidation degree to ensure sufficient edge-to-edge connection. (c) Mechanical property summary of the graphene fibers using GO sheets of different oxidation degree and their relationship with amine concentrations.

With increase in oxidation time, the XPS C-C/C=C signals decrease while those of C-OH/C-O-C and carboxyl increase. For oxidation times greater than 2.25 h, the fraction of C-OH/C-O-C becomes dominant and a slight shift to higher energy of the C-C/C=C peaks occurred, which could be due to the significant increase in C-C. Raman spectra also showed increase in D band for the sample treated for longer than 2.25 h. We believe that the degradation of mechanical properties beyond 2.25 h is mainly due to the destruction of the planar graphene structure.

Irrespective of whether the oxidation degree of the GO sheets was low or very high (with in-plane defects), the fibers with amide linking protocol showed improved performances compared to the corresponding control fiber with no linking, validating the efficiency of the proposed strategy.

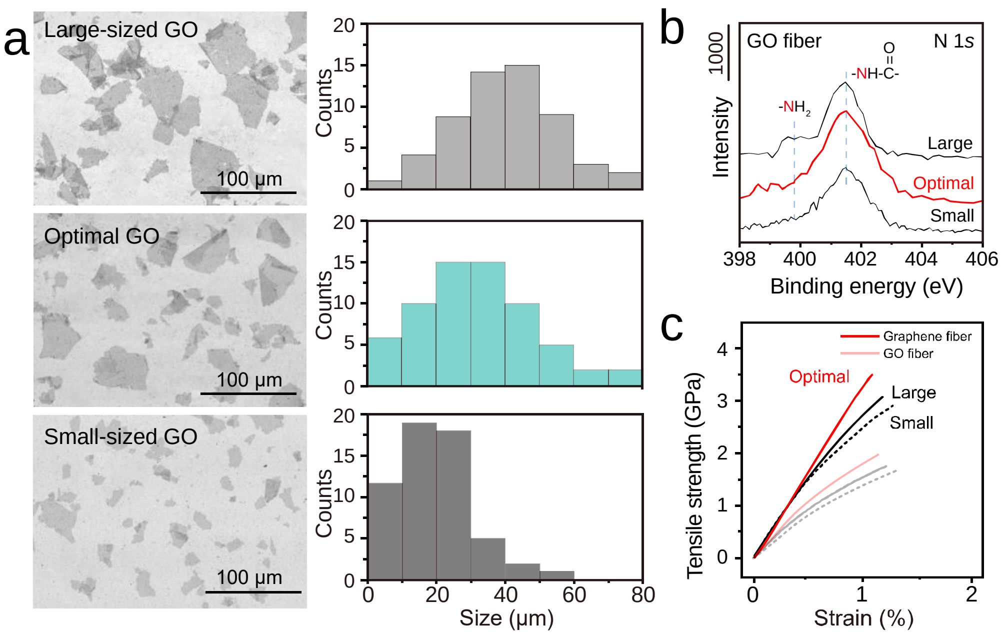

Supplementary Figure 39. Mechanical performance comparison of fibers prepared with GO of differing sizes.

(a) SEM images and size distribution for GO of different lateral sizes. The size sieving was done by centrifugation; 2000–4000 rpm (434–1735 $\times g$) for large sheets and >6000 rpm (3904 $\times g$) for small sheets. (b) XPS N 1*s* data of the fibers, (c) Comparison of tensile strengths of fibers obtained from GO of different lateral sizes.

Selectively sieving large sheets did not bring about better mechanical performances. N 1*s* spectrum for fiber from exclusively large sheets shows the obvious presence of remnant $–NH_2$ and weaker signal of amide, suggesting incomplete patching of GO sheets probably due to steric effect; fibers from exclusively small sheets may have more defects due to the larger number of boundaries.

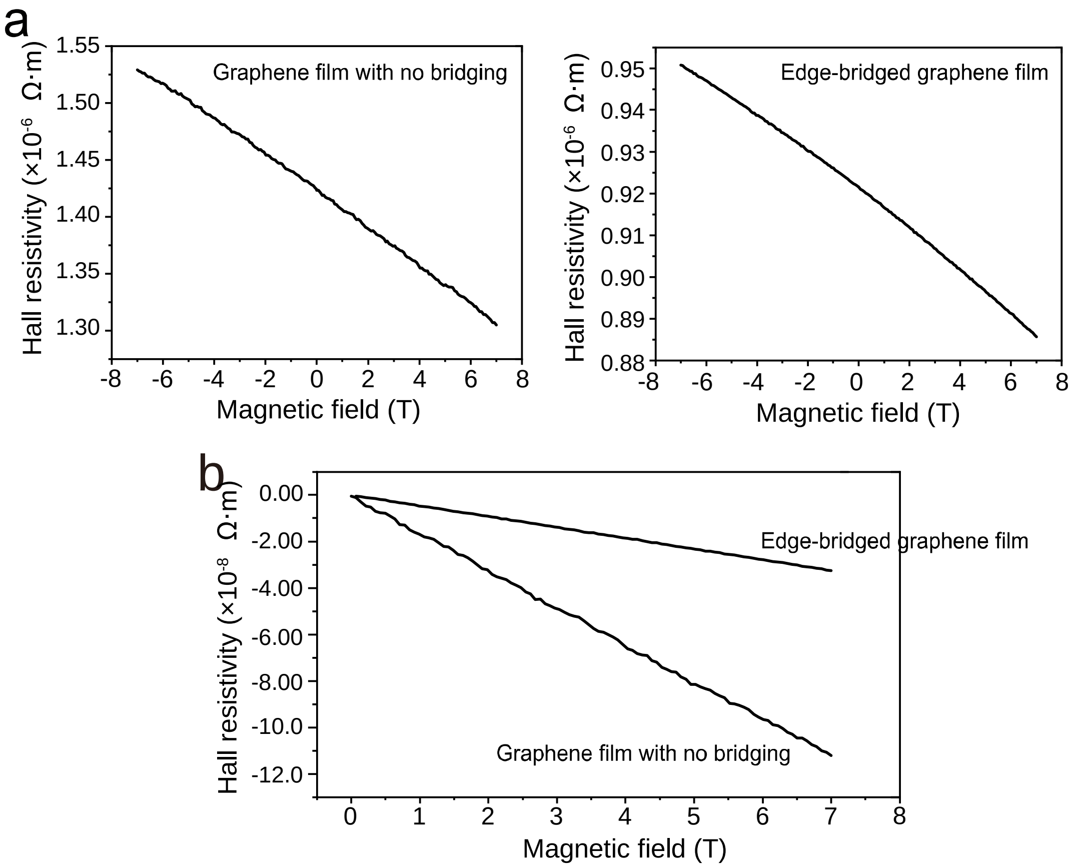

Supplementary Figure 40. Examination of the origin of improvement in electrical conductivity by Hall effect measurements.

Magnetic field (B) dependence of Hall resistivity ρ_{yx} at room temperature of an edge-bridged graphene film compared with a film with no bridging, (a) before and (b) after antisymmetrization procedure of the transverse voltage drops, $\rho_{yx} = \frac{\rho_{yx}(+B)-\rho_{yx}(-B)}{2}$.

The standard ac lock-in techniques (~113 Hz) were used by applying the magnetic field in opposite directions (parallel and anti-parallel to the c axis). Based on the equations $R_{\mathrm{H}} = \frac{t}{I} \cdot \frac{V}{B} = \frac{\rho_{yx}}{B}$ and $n = \frac{1}{|R_{\mathrm{H}}| \cdot q}$, the carrier concentration (n) is inversely proportional to the Hall coefficient (R_{H}) or the slope of plot ρ_{yx}-B. From these data, it can be concluded that the carrier density was greatly improved (~ 5 times) for the edge-bridged film.

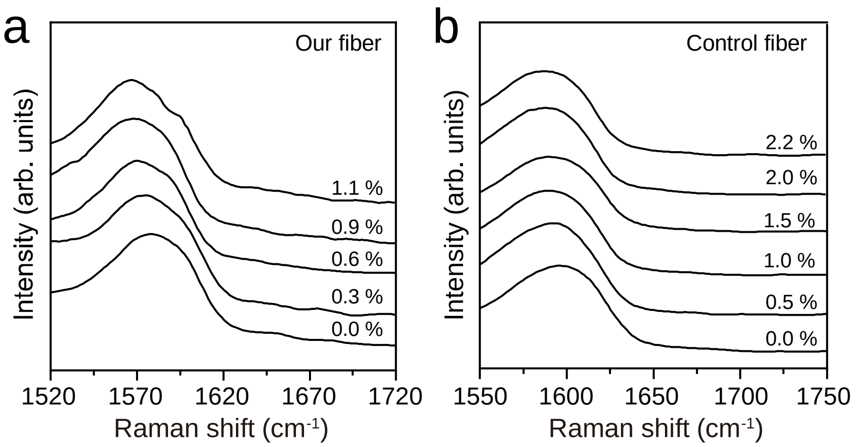

Supplementary Figure 41. In-situ Raman spectra collected at different external strains.

In-situ Raman spectra during loading different external strains on (a) amide-connected graphene fiber and (b) control graphene fiber obtained by solvent exchange.

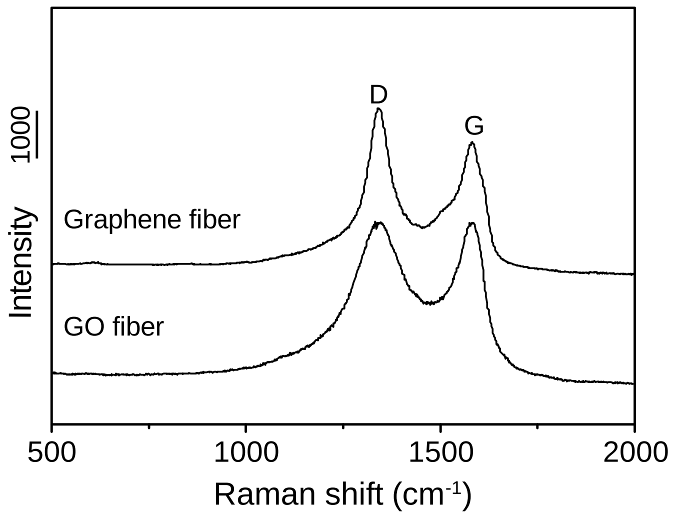

Supplementary Figure 42. Raman spectra of graphene fiber after chemical reduction compared with the corresponding precursor GO fiber.

The Raman band at 1585 cm^{-1} is related to C–C vibrations in graphite, and the signal at 1330 cm^{-1} arises from the boundaries of the graphite crystals and disorder in the sp^2-hybridized carbon system. The two bands are denoted as the G and D bands, respectively. The intensity of the D-band is still quite strong for the graphene assembly after chemical reduction. This result could be due to incomplete restoration of the defects created during elimination of oxygen-containing groups or structural disorder created during the reduction process. Thus, there is plenty of room to further improve the overall mechanical and physical properties of the assembly.

Supplementary Table 1. Gravimetric density of graphene fibers prepared in this work along with reported values in the literature for other graphene fibers. The theoretical value for graphite is 2.2 g cm^{-3}.

Sample	Density (g cm^{-3})	
	**Chemical reduction**	**Thermal reduction**
**Our fiber with edge connection**	**1.90**	/
**Control fiber without edge connection via solvent exchange**	**1.72**	/
Plasticization spinning of graphene fiber[10] (solvent exchange)	1.75	1.90 @ 2800 °C
Graphene nanoribbon-based graphene fiber[11]	/	0.88 @ 1500 °C
Glass pipeline-assisted graphene fiber[12]	/	0.23 @ 230 °C
Concentrated GO wet-spun graphene fiber[13]	0.31	/
Large/small nanosheets optimized graphene fiber[14] ($CaCl_2$)	/	1.88 @ 2800 °C
Liquid crystal self-templated graphene fiber[15] ($CaCl_2$)	1.00	/

Supplementary Table 2. Mechanical performance of graphene fibers produced in this work along with reported values in the literature for other graphene fibers.

Sample	Tensile strength (GPa)	Young's modulus (GPa)
Graphene fiber after chemical reduction		
**Our fiber with edge connection**	**3.54 ± 0.25**	**340 ± 32**
**Control fiber without edge connection via solvent exchange**	**1.9 ± 0.1**	**127 ± 24**
Plasticization spinning of graphene fiber[10] (solvent exchange)	2.2	~180
Concentrated GO wet-spun graphene fiber[13]	0.36	12.8
Chiral liquid crystal spun graphene fiber[16]	0.14	7.7
Macroscopic graphene fiber[17]	0.18	8.7
Crosslinking reinforced graphene fiber[18] (PDA)	0.23	5.9
Liquid crystal self-templated graphene fiber[15] ($CaCl_2$)	0.49	17.6
Hydrogel drawing graphene fiber[19] ($CaCl_2$, ammonia)	0.30	38.3
Giant GO sheets assembled graphene fiber[20] ($CaCl_2$, $CuSO_4$)	0.50	11.2
Graphene fiber after thermal reduction		
Plasticization spinning of graphene fiber[10] (solvent exchange)	3.4	342 @ 2800 ℃
Microfluidic assembled graphene fiber[21] (solvent exchange)	1.9	309 @ 2500 ℃
Synergetic defect engineering graphene fiber[1] (solvent exchange)	2.2	400 @ 3000 ℃
Graphene nanoribbon-based graphene fiber[11]	0.38	39.9 @ 1050 ℃
Crosslinking reinforced graphene fiber[18] (PDA)	0.72	48.6 @ 1200 ℃
Large/small nanosheets optimized graphene fiber[14] ($CaCl_2$)	1.2	143 @ 2800 ℃

PDA: polydopamine.

Supplementary Table 3. Electrical conductivity of graphene fibers prepared in this work along with reported values in the literature for other graphene fibers.

Sample	Electrical conductivity (S m^{-1})
Graphene fiber after chemical reduction	
**Our fiber with edge connection**	**1.5×10^5**
**Control fiber without edge connection via solvent exchange**	**0.32×10^5**
Plasticization spinning of graphene fiber[10] (solvent exchange)	0.30×10^5
Concentrated GO wet-spun graphene fiber[13]	0.32×10^5
Chiral liquid crystal spun graphene fiber[16]	0.25×10^5
Macroscopic neat graphene fiber[17]	0.03×10^5
Crosslinking reinforced graphene fiber[18] (PDA)	0.66×10^5
Liquid crystal self-templated graphene fiber[15] ($CaCl_2$)	0.05×10^5
Giant GO sheets assembled graphene fiber[20] ($CaCl_2$, $CuSO_4$)	0.41×10^5
Graphene fiber after thermal reduction	
Plasticization spinning of graphene fiber[10] (solvent exchange)	1.2×10^6 @ 2800 °C
Microfluidic assembled graphene fiber[21] (solvent exchange)	1.0×10^6 @ 2500 °C
Synergetic defect engineering graphene fiber[1] (solvent exchange)	8.0×10^5 @ 3000 °C
Graphene nanoribbon-based graphene fiber[11]	0.28×10^5 @ 1500 °C
Large/small nanosheets optimized graphene fiber[14] ($CaCl_2$)	2.2×10^5 @ 2800 °C

PDA: polydopamine.

References

1. Xu, Z., Liu, Y., Zhao, X., Li, P., Sun, H., Xu, Y., Ren, X., Jin, C., Xu, P., Wang, M. & Gao, C. Ultrastiff and strong graphene fibers via full-scale synergetic defect engineering. *Adv. Mater.* **28**, 6449-6456 (2016).
2. Patel, K., Gayakwad, E. & Shankarling, G. Graphene oxide as a metal-free carbocatalyst for direct amide synthesis from carboxylic acid and amine under solvent-free reaction condition. *ChemistrySelect* **5**, 8295-8300 (2020).
3. Tutorial video at https://www.youtube.com/watch?v=buRFisuSJCM
4. Fan, X., Peng, W., Li, Y., Li, X., Wang, S., Zhang, G. & Zhang, F. Deoxygenation of exfoliated graphite oxide under alkaline conditions: a green route to graphene preparation. *Adv. Mater.* **20**, 4490-4493 (2008).
5. Hung, W., Tsou, C., De Guzman, M., An, Q., Liu, Y., Zhang, Y., Hu, C., Lee, K. & Lai, J. Cross-linking with diamine monomers to prepare composite graphene oxide-framework membranes with varying *d*-Spacing. *Chem. Mater.* **26**, 2983-2990 (2014).
6. Jia, Z., Wang, Y., Shi, W. & Wang, J. Diamines cross-linked graphene oxide free-standing membranes for ion dialysis separation. *J. Membr. Sci.* **520**, 139-144 (2016).
7. Qian, Y., Zhou, C. & Huang, A. Cross-linking modification with diamine monomers to enhance desalination performance of graphene oxide membranes. *Carbon* **136**, 28-37 (2018).
8. Woo, J., Oh, J., Jo, S. & Han, C. Nacre-mimetic graphene oxide/cross-linking agent composite films with superior mechanical properties. *ACS Nano* **13**, 4522-4529 (2019).
9. Dimiev, A., Alemany, L. & Tour, J. Graphene oxide. origin of acidity, its instability in water, and a new dynamic structural model. *ACS Nano* **7**, 576-588 (2013).
10. Li, P., Liu, Y., Shi, S., Xu, Z., Ma, W., Wang, Z., Liu, S. & Gao, C. Highly crystalline graphene fibers with superior strength and conductivities by plasticization spinning. *Adv. Funct. Mater.* **30**, 52, 2006584 (2020).
11. Xiang, C., Behabtu, N., Liu, Y., Chae, H., Young, C., Genorio, B., Tsentalovich, D., Zhang, C., Kosynkin, D., Lomeda, J., Hwang, C., Kumar, S., Pasquali, M. & Tour, J. Graphene nanoribbons as an advanced precursor for making carbon fiber. *ACS Nano* **7**, 1628−1637 (2013).
12. Dong, Z., Jiang, C., Cheng, H., Zhao, Y., Shi, G., Jiang, L. & Qu, L. Facile fabrication of light, flexible and multifunctional graphene fibers. *Adv. Mater.* **24**, 1856−1861 (2012).

13. Chen, L., He, Y., Chai, S., Qiang, H., Chen, F. & Fu, Q. Toward high performance graphene fibers. *Nanoscale* **5**, 5809 (2013).
14. Xin, G., Yao, T., Sun, H., Scott, S., Shao, D., Wang, G. & Lian, J. Highly thermally conductive and mechanically strong graphene fibers. *Science* **349**, 1083−1087 (2015).
15. Hu, X., Xu, Z., Liu, Z. & Gao, C. Liquid crystal self-templating approach to ultrastrong and tough biomimic composites. *Sci. Rep.* **3**, 2374 (2013).
16. Xu, Z. & Gao, C. Graphene chiral liquid crystals and macroscopic assembled fibres. *Nat. Commun.* **2**, 571 (2011).
17. Cong, H., Ren, X., Wang, P. & Yu, S. Wet-spinning assembly of continuous, neat, and macroscopic graphene fibers. *Sci. Rep.* **2**, 613 (2012).
18. Ma, T., Gao, H., Cong, H., Yao, H., Wu, L., Yu, Z., Chen, S. & Yu, S. A bioinspired interface design for improving the strength and electrical conductivity of graphene-based fibers. *Adv. Mater.* **30**, 1706435, (2018).
19. Park, H., Lee, K., Kim, Y., Ambade, S., Noh, S., Eom, W., Hwang, J., Lee, W. & Huang, J., Han, T. Dynamic assembly of liquid crystalline graphene oxide gel fibers for ion transport. *Sci. Adv.* **4**, 11(2018).
20. Xu, Z., Sun, H., Zhao, X. & Gao, C. Ultrastrong fibers assembled from giant graphene oxide sheets. *Adv. Mater.* **25**, 188–193 (2013).
21. Xin, G., Zhu, W., Deng, Y., Cheng, J., Zhang, L., Chung, A., De, S. & Lian, J. Microfluidics-enabled orientation and microstructure control of macroscopic graphene fibres. *Nat. Nanotech.* **14**, 168−175 (2019).
