# Supplementary material for: Covalently bridging graphene edges for improving mechanical and electrical properties of fibers
Source: Nat Commun. 2024 Jun 7;15:4880. doi: 10.1038/s41467-024-49270-5 (PMC11161649; doi:10.1038/s41467-024-49270-5)
Supplement: Supplementary file 3 — Reporting Summary [file 41467_2024_49270_MOESM3_ESM.pdf]

nature portfolio

Corresponding author(s): NCOMMS-23-52701B

Last updated by author(s): Apr 30, 2024

Reporting Summary

Nature Portfolio wishes to improve the reproducibility of the work that we publish. This form provides structure for consistency and transparency in reporting. For further information on Nature Portfolio policies, see our Editorial Policies and the Editorial Policy Checklist.

Statistics

For all statistical analyses, confirm that the following items are present in the figure legend, table legend, main text, or Methods section.

n/a	Confirmed	
☐	☒	The exact sample size (n) for each experimental group/condition, given as a discrete number and unit of measurement
☐	☒	A statement on whether measurements were taken from distinct samples or whether the same sample was measured repeatedly
☐	☒	The statistical test(s) used AND whether they are one- or two-sided<br>*Only common tests should be described solely by name; describe more complex techniques in the Methods section.*
☐	☒	A description of all covariates tested
☐	☒	A description of any assumptions or corrections, such as tests of normality and adjustment for multiple comparisons
☐	☒	A full description of the statistical parameters including central tendency (e.g. means) or other basic estimates (e.g. regression coefficient) AND variation (e.g. standard deviation) or associated estimates of uncertainty (e.g. confidence intervals)
☐	☒	For null hypothesis testing, the test statistic (e.g. F, t, r) with confidence intervals, effect sizes, degrees of freedom and P value noted<br>*Give P values as exact values whenever suitable.*
☐	☒	For Bayesian analysis, information on the choice of priors and Markov chain Monte Carlo settings
☐	☒	For hierarchical and complex designs, identification of the appropriate level for tests and full reporting of outcomes
☐	☒	Estimates of effect sizes (e.g. Cohen's d, Pearson's r), indicating how they were calculated

Our web collection on statistics for biologists contains articles on many of the points above.

Software and code

Policy information about availability of computer code

Data collection	no software was used
Data analysis	no software was used

For manuscripts utilizing custom algorithms or software that are central to the research but not yet described in published literature, software must be made available to editors and reviewers. We strongly encourage code deposition in a community repository (e.g. GitHub). See the Nature Portfolio guidelines for submitting code & software for further information.

Data

Policy information about availability of data

All manuscripts must include a data availability statement. This statement should provide the following information, where applicable:

- Accession codes, unique identifiers, or web links for publicly available datasets
- A description of any restrictions on data availability
- For clinical datasets or third party data, please ensure that the statement adheres to our policy

All data that support the findings of this study are available in the main article and Supplementary Information.

Research involving human participants, their data, or biological material

Policy information about studies with human participants or human data. See also policy information about sex, gender (identity/presentation), and sexual orientation and race, ethnicity and racism.

Reporting on sex and gender	this information is not collected
Reporting on race, ethnicity, or other socially relevant groupings	no report on rare, ethnicity, or other socially relevant groupings
Population characteristics	no related information in the work
Recruitment	no related information in the work
Ethics oversight	no related information in the work

Note that full information on the approval of the study protocol must also be provided in the manuscript.

Field-specific reporting

Please select the one below that is the best fit for your research. If you are not sure, read the appropriate sections before making your selection.

☐ Life sciences ☐ Behavioural & social sciences ☒ Ecological, evolutionary & environmental sciences

For a reference copy of the document with all sections, see nature.com/documents/nr-reporting-summary-flat.pdf

Ecological, evolutionary & environmental sciences study design

All studies must disclose on these points even when the disclosure is negative.

Study description	The work is a study to bridge graphene edge to improve mechanical and electrical properties of macroscopic fiber assembly. The details for property measurement and possible factors that affect the measurement are described in SI.
Research sample	We continuously injected the GO colloid into a coagulation bath to achieve the sample. In the sample choice for the following measurement, actually we could not tell if the macroscopic performance is good or not just by checking the appearance, but we may not use the parts at the two ends.
Sampling strategy	As we also described in SI, samples with gauge length of 20 mm were used for property measurement. The length was determined by a ruler. The gauge length was set based on previous references.
Data collection	As described in authors contributions, L. D., T. X., J. Z., J. J., and Z. S. performed materials synthesis and characterization studies. Y. X., T. L. helped with FIB cutting and data collection on STEM-in-SEM studies.
Timing and spatial scale	The data collection started from May, 2020 until submission of the revision, March, 2024. We have been supplementing more data into the work. We collected data for the initial manuscript organization, and then later data collection was also performed in manuscript revisions.
Data exclusions	In the tensile measurement, the sample may be loosened and the data may be much deviated, which were excluded.
Reproducibility	For all the experiments, reproducibility was confirmed, by repeating at least 10 times independently. And in the long submission process, the student who was in the main duty switched 3 times. Each time, the new student would re-do the previous experiments, and all of the repeating experiments were finally successful.
Randomization	For samples synthesized from the same conditions, we selected samples just by random with no group allocation.
Blinding	In data acquisition and analysis, we used standard procedures for each specific measurement.

Did the study involve field work? ☐ Yes ☒ No

Reporting for specific materials, systems and methods

We require information from authors about some types of materials, experimental systems and methods used in many studies. Here, indicate whether each material, system or method listed is relevant to your study. If you are not sure if a list item applies to your research, read the appropriate section before selecting a response.

Materials & experimental systems

n/a	Involved in the study
☒	☐ Antibodies
☒	☐ Eukaryotic cell lines
☒	☐ Palaeontology and archaeology
☒	☐ Animals and other organisms
☒	☐ Clinical data
☒	☐ Dual use research of concern
☒	☐ Plants

Methods

n/a	Involved in the study
☒	☐ ChIP-seq
☒	☐ Flow cytometry
☒	☐ MRI-based neuroimaging

Plants

Seed stocks	*Report on the source of all seed stocks or other plant material used. If applicable, state the seed stock centre and catalogue number. If plant specimens were collected from the field, describe the collection location, date and sampling procedures.*
Novel plant genotypes	*Describe the methods by which all novel plant genotypes were produced. This includes those generated by transgenic approaches, gene editing, chemical/radiation-based mutagenesis and hybridization. For transgenic lines, describe the transformation method, the number of independent lines analyzed and the generation upon which experiments were performed. For gene-edited lines, describe the editor used, the endogenous sequence targeted for editing, the targeting guide RNA sequence (if applicable) and how the editor was applied.*
Authentication	*Describe any authentication procedures for each seed stock used or novel genotype generated. Describe any experiments used to assess the effect of a mutation and, where applicable, how potential secondary effects (e.g. second site T-DNA insertions, mosiacism, off-target gene editing) were examined.*
